# Japanese-Style Dietary Patterns, Mortality, and Selected Cancer Outcomes in Japanese Populations: A Systematic Review Integrating Meta-Analysis, Food-Group Loading Visualization, and Qualitative Synthesis

**DOI:** 10.1016/j.advnut.2026.100756

**Published:** 2026-10-15

**Authors:** Tao Yu, Marina Yamagishi, Kota Okoshi, Shuhei Nomura, Naoko Yamamoto, Shoichiro Tsugane

**Affiliations:** 1 Department of Food, Nutrition and Health, Graduate School of Public Health, International University of Health and Welfare, Tokyo, Japan; 2 R&D Division, Meiji Co., Ltd., Tokyo, Japan; 3 Wellness Solution Development Center, Institute of Food Sciences and Technologies, Ajinomoto Co., Inc., Kanagawa, Japan; 4 Department of Health Policy and Management, School of Medicine, Keio University, Tokyo, Japan; 5 Global Health Policy Lab, International Research Institute of Disaster Science, Tohoku University, Miyagi, Japan

**Keywords:** Japanese diet, Washoku, Japanese Diet Index, a priori dietary patterns, a posteriori dietary patterns, food structure, meta-analysis, cardiovascular mortality, gastric cancer, colorectal cancer

## Abstract

Japanese-style dietary patterns (DPs) are often regarded as healthy, but the food-group combinations needed to develop health-oriented Japanese-style DPs remain unclear. This systematic review synthesized associations between predefined (a priori) Japanese-style DPs and health outcomes in Japanese populations and used data-derived (a posteriori) DPs to examine outcome-specific heterogeneity in food-group composition and loadings. PubMed/MEDLINE and Web of Science Core Collection were searched for observational studies. Random-effects meta-analyses were conducted for comparable a priori Japanese-style DPs using adjusted hazard ratios (HRs) comparing the highest and lowest score categories. A posteriori DPs derived by principal component analysis or factor analysis were visualized using reported estimates, investigator-assigned pattern names, relative similarity to the Japanese Diet Index-9 (JDI-9) reference profile, and food-group loadings. Of 37 reports retained in the review, 12 longitudinal reports contributed to meta-analyses or food-group loading visualizations, and 25 were synthesized qualitatively. Across 3 independent studies contributing 5 analytic estimates per outcome, pooled HRs comparing the highest and lowest score categories suggested lower all-cause mortality [HR: 0.88; 95% confidence interval (CI): 0.83, 0.93; *I*^2^ = 61.1%) and cardiovascular disease mortality (HR: 0.82; 95% CI: 0.73, 0.93; *I*^2^ = 71.6%), although heterogeneity was substantial; the pooled estimate for total cancer mortality was close to the null. Among 43 a posteriori DPs from 9 studies, 21 were classified as JDI-9-like; however, this relative classification alone did not consistently distinguish reported associations across outcomes; interpretation required examination of the reported component-level food-group loadings. These findings motivate a provisional 3-layer framework distinguishing potentially favorable traditional components, high-sodium traditional components, and contemporary dietary components; the framework is hypothesis-generating and requires outcome-specific validation.

This review protocol was registered at PROSPERO as CRD420261322284.

**Statement of significance**

This review advances research on Japanese-style dietary patterns by integrating pooled evidence on a priori patterns with food-group loading visualization of a posteriori patterns. Pooled estimates from 3 independent studies suggested lower all-cause and cardiovascular disease mortality with greater alignment with a priori patterns, whereas the visualizations indicated that Japanese Diet Index-9 similarity alone did not consistently distinguish associations with selected health outcomes and that interpretation requires examination of outcome-specific food-group loadings.

## Introduction

Japanese-style dietary patterns (DPs) have long been discussed in relation to health, longevity, and nutritional epidemiology, but their definition and health outcome evidence remain difficult to synthesize [1], [2], [3], [4], [5]. They are commonly characterized by dietary structures that include rice, seafood, soy products, vegetables, seaweed, miso soup, pickles, and green tea and have often been discussed in relation to Japanese longevity and historical changes in cardiovascular disease (CVD) mortality [6]. In this review, “food structure” refers to the pattern-level configuration of food groups, including the groups represented, their scoring direction in a priori DPs, and the direction and magnitude of their loadings in a posteriori DPs; it does not refer to the physical structure, texture, or food matrix of individual foods. Studies of the Japanese Food Guide Spinning Top and meals combining staple, main, and side dishes suggest that such patterns can support food-group diversity and nutrient adequacy [7], [8], [9], [10], [11], [12], [13]. However, potentially favorable components coexist with sodium-rich foods and seasonings, for which guidance-based indicators have yielded inconsistent findings [9], [10], [13], [14], [15], [16], [17], [18], [19]. Japanese-style DPs should therefore be evaluated as whole patterns.

A qualitative systematic review identified 33 characteristics of the Japanese diet across 16 food categories, illustrating both recurring features and substantial variation in epidemiologic operationalization [20]. Unlike Mediterranean diet research, Japanese-style DP research lacks a widely accepted scoring framework and empirically calibrated absolute intake criteria that are comparable across studies, limiting evidence synthesis and international comparison [21], [22], [23].

Evidence can be organized into 2 complementary approaches [24], [25]. Predefined (a priori) DPs specify food components and scoring directions in advance and are relatively amenable to meta-analysis, although study-specific intake thresholds limit cross-study comparability [26], [27]. Previous prospective evidence has suggested lower CVD mortality with greater alignment with these scores [6]. Thus, evidence based on a priori Japanese-style DPs has accumulated particularly for mortality outcomes, especially CVD mortality. The second consists of data-derived (a posteriori) DPs extracted from intake data using methods such as principal component analysis (PCA) or factor analysis [28]. This approach can complement a priori evidence because it captures dietary structures as they occur within study populations [29]. However, pattern names are assigned by investigators and do not necessarily denote similar food structures; food-group loadings define the exposure more directly. Murakami et al. [30] systematically reviewed PCA-derived DPs in Japanese adults and showed that 285 extracted DPs could be broadly organized into 6 recurring major DPs. The concept of the Japanese diet is also dynamic rather than static and responsive to changes over time [31], [32], [33].

Against this background, this review synthesized evidence on Japanese-style DPs and health outcomes in Japanese populations. A priori scores were meta-analyzed when comparable estimates were available, whereas a posteriori DPs were descriptively visualized using reported associations, investigator-assigned names, Japanese Diet Index-9 (JDI-9) similarity, and food-group loadings. Reports not contributing to these analytic syntheses were summarized qualitatively.

## Methods

### Protocol, search strategy, and eligibility criteria

The review protocol was registered in PROSPERO on 24 February 2026 (CRD420261322284). The preregistered main outcomes were all-cause mortality, CVD, stroke, cancer, long-term care need, decline in activities of daily living or instrumental activities of daily living, cognitive function, falls or fractures, sarcopenia, and frailty. No secondary outcome hierarchy was prespecified.

PubMed/MEDLINE was selected for its coverage of biomedical and nutrition science literature, and Web of Science Core Collection for its broader multidisciplinary coverage and citation indexing. Both databases were searched from inception through 14 March 2026; the initial searches were conducted on 24 February 2026 and updated on 14 March 2026. The strategies combined Japanese-style dietary exposure terms, outcome terms based on the preregistered scope, and study-design terms. PubMed used title/abstract terms and MeSH headings; Web of Science used the Topic field with the Document Types filter restricted to article (Supplemental Methods). The reference lists of reports contributing to the meta-analyses or food-group loading visualizations were also manually screened for additional eligible reports.

The registered eligibility criteria covered nonrandomized cross-sectional or cohort studies of general community-dwelling populations residing in Japan, without age restrictions and not selected on the basis of a target disease. Eligible exposures were predefined Japanese-style DP scores or data-derived Japanese(-like) DPs. Review eligibility and synthesis-specific requirements are summarized in Supplemental Table 4.

After study selection, reports were assessed for meta-analysis, food-group loading visualization, or qualitative synthesis (Supplemental Table 4). The protocol limited the meta-analysis to cohort studies with sufficiently comparable evidence. Additional analytic requirements were specified during data extraction, before the final synthesis. Longitudinal studies were used for meta-analysis and visualization to preserve temporal ordering. Meta-analyses required comparable a priori scores and adjusted hazard ratios (HRs) with 95% confidence intervals (CIs) comparing the highest and lowest score categories. Visualizations required PCA- or factor-analysis–derived patterns with category-based estimates and sufficient food-group loadings for reconstruction and comparison. Other retained reports were synthesized qualitatively.

For the a priori meta-analyses, when multiple publications, irrespective of authorship, reported the same outcome from the same cohort using the same exposure definition approach, only the most recent publication contributed to the pooled estimate. The earlier overlapping report remained in the qualitative synthesis. Cohort overlap did not, by itself, exclude a posteriori reports from descriptive visualization, and these reports were not treated as independent cohorts in a meta-analysis.

#### Exposure definitions and JDI-9 similarity assessment

Exposures were classified into 2 categories: a priori Japanese-style DPs and a posteriori DPs. First, a priori Japanese-style DPs were defined as dietary exposures that explicitly quantified alignment with predefined Japanese-style food structures. This category included the JDI family, modified JDI scores, and related Japanese-style DP scores.

For the present food-group loading visualizations, a posteriori DPs were restricted to those derived from dietary intake data using PCA or factor analysis. Investigator-assigned pattern names were retained for display but were not used to determine JDI-9 similarity. The JDI-9 was selected as the reference because it was the most frequently used a priori Japanese-style index among the eligible studies. To characterize similarity to the JDI-9 reference profile in a reproducible manner, we represented each a posteriori DP as an ordered 9-component vector of reported food-group loadings mapped to the JDI-9 components. The JDI-9 reference profile comprised 7 positive scoring items—rice, miso or miso soup, fish and shellfish, vegetables, seaweed, pickles, and green tea—and 2 negative scoring items—meat and coffee. These terms indicate scoring direction and do not imply beneficial or harmful health effects. When an original food group corresponded directly to a JDI-9 component, such as rice, fish and shellfish, seaweed, pickles, green tea, meat, or coffee, the reported loading was assigned to the corresponding component. When multiple original food groups corresponded to one JDI-9 component, such as green and yellow vegetables and other vegetables for the vegetable component, their loadings were averaged. Mapping decisions were made with reference to the original food-group definitions, previous work on Japanese PCA-derived DPs, and Japanese dietary habits [30]. The original loading scales and signs reported in each study were retained; no rescaling or sign reversal was applied.

When a food group had been assessed in the original study but was not shown in the reported loading table, generally because the loading was below the study-specific reporting threshold, its loading was assigned a value of 0 for harmonization. When the relevant food group had not been assessed in the original dietary assessment instrument or could not be mapped from the original reporting scheme, the corresponding JDI-9 component was treated as unavailable and excluded from the similarity calculations. The number of available JDI-9 components was recorded for each DP and is shown in Supplemental Table 3. Using these reconstructed JDI-9 component vectors, we assessed similarity to the JDI-9 reference profile using 2 complementary metrics. First, cosine similarity was calculated by comparing each reconstructed vector with the JDI-9 reference vector, in which the 7 positive scoring items were assigned +1 and the 2 negative scoring items were assigned −1. This directional coding was used only for the similarity calculations. Higher cosine similarity scores indicate greater similarity to the JDI-9 reference profile. Second, a distance score was calculated as the mean absolute distance between the reconstructed loading and the corresponding JDI-9 reference value across available components. Lower distance scores indicate greater similarity to the JDI-9 reference profile.

For each metric, a posteriori DPs were categorized into quartiles according to their relative position among all classifiable a posteriori DPs. For cosine similarity, quartiles were labeled from highest to lowest similarity as Closest, Close, Far, and Farthest. For the distance score, quartiles were labeled from lowest to highest distance using the same terms. In the absence of an externally validated cutoff, the Closest and Close categories were used as an exploratory distribution-based threshold. To assess concordance between the 2 metric-specific ordinal classifications, the categories were coded as Farthest = 1, Far = 2, Close = 3, and Closest = 4, and Spearman’s rank correlation coefficient was calculated. Because cosine similarity captures directional agreement with the JDI-9 reference profile, whereas the distance score captures component-wise distance from the reference values on the retained loading scale, the primary binary classification used a conservative conjunctive rule. A posteriori DPs classified as Closest or Close by both metrics were defined as JDI-9-like DPs; all other DPs were labeled non–JDI-9-like for binary visualization, indicating that they did not meet the primary operational definition. To assess the robustness of this combination rule, we also repeated the binary labeling using the Closest or Close criterion for cosine similarity alone and for the distance score alone. The classification was used as a relative food-structural annotation for visualization and interpretation, not as a validated definition of a healthful DP.

### Study selection, data extraction, statistical analysis, visualization, and risk-of-bias assessment

Three reviewers with doctoral-level training in nutritional epidemiology or public health (TY, MY, and KO) independently screened titles and abstracts, assessed full texts, and extracted the prespecified data items from each included report. The reviewers then compared their screening decisions and extracted data in a face-to-face meeting. Discrepancies were resolved by consensus. Extracted information included the first author, publication year, cohort or study name, setting, study design, sample size, age, sex, dietary assessment method, exposure definition, outcome, follow-up duration, effect measure, adjustment factors, study funding source, investigator-assigned pattern names, and food-group loadings where applicable.

Effect estimates were transformed into the natural logarithm scale, and SEs were calculated from reported 95% CIs. Sex-specific estimates were treated as separate analytic strata only when an overall estimate was not reported. Because DP score ranges and category boundaries differed across studies, study-specific highest and lowest score categories were compared. For a priori Japanese-style DPs, meta-analyses focused on all-cause mortality, CVD mortality, and total cancer mortality. Random-effects meta-analyses were conducted only for a priori Japanese-style DPs, with between-study variance estimated using restricted maximum likelihood. Heterogeneity was assessed using the *I*^2^ statistic and tests for heterogeneity. *I*^2^ values were interpreted using the approximate, overlapping ranges of 0%–40% as potentially unimportant, 30%–60% as moderate, 50%–90% as substantial, and 75%–100% as considerable.

Longitudinal studies of a posteriori DPs reporting all-cause mortality, CVD mortality, gastric cancer incidence or mortality, or colorectal cancer incidence were presented as outcome-specific food-group loading visualizations. For each a posteriori DP, the visualization combined the reported effect estimate and 95% CI, the investigator-assigned pattern name, and the sex stratum, where applicable, JDI-9-like classification, and component food-group loadings. These visualizations supported component-level interpretation and hypothesis generation.

Reports not contributing to the meta-analyses or loading visualizations were tabulated and synthesized narratively by outcome domain (Supplemental Table 6). Study designs, a priori and data-derived patterns, and score levels and changes were distinguished, whereas reported models, comparisons, sex strata, effect estimates, and uncertainty intervals were preserved. Reports evaluating both pattern approaches were listed once.

Risk of bias was assessed for longitudinal reports in both the analytic and qualitative syntheses using the Risk Of Bias In Nonrandomized Studies of Exposures (ROBINS-E) framework described by Higgins et al. [34]. The assessed domains were confounding, measurement of the exposure, selection of participants into the study or analysis, postexposure interventions, missing data, measurement of the outcome, and selection of the reported result. The domain-specific operational criteria were adapted from Wallerer et al. [35] and modified for the present review of DP studies (Supplemental Table 1).

For exposure measurement, potential nondifferential dietary measurement error was considered in accordance with established nutritional-epidemiologic principles [36], and the absence of repeated dietary measurements and possible changes in habitual diet during follow-up were considered under Domain 2. Domain 4 was restricted to postexposure interventions that were influenced by prior dietary exposure and could affect the outcome. For longitudinal reports, the overall judgment corresponded to the highest risk-of-bias judgment across applicable domains. For qualitatively synthesized reports, the model or result in Supplemental Table 6 was fixed as the assessment target, and bias was judged for interpretation of the overall DP effect. When 2 adjacent judgments remained plausible after source review, the higher-risk category was assigned; insufficient information was retained as “No information.” Cross-sectional reports received review-specific assessments of applicable domains, with Domain 4 and the overall judgment shown as “-” (Supplemental Table 7). All statistical analyses were performed using Stata version 18.0 (StataCorp LLC).

## Results

### Study selection and study characteristics

The study selection process is summarized in Figure 1. The searches identified 1362 records; after duplicate removal, 1184 records remained, and 46 reports underwent full-text assessment. Nine reports were excluded at this stage; the excluded reports, their primary Population, Exposure, Comparator, Outcome, Timing, and Setting (PECOTS) domains, and the primary reasons for exclusion are detailed in Supplemental Table 5. Thirty-seven reports were retained: 12 longitudinal reports contributed to the analytic syntheses and 25 were included in qualitative synthesis only (Supplemental Tables 6 and 7). Manual screening of the 12 analytic reports’ reference lists identified no additional eligible reports. Three a priori DP studies contributed to the main meta-analyses [37], [38], [39], and 9 a posteriori DP studies contributed to the auxiliary food-group loading visualizations [40], [41], [42], [43], [44], [45], [46], [47], [48]. Outcomes included all-cause mortality, CVD mortality, total cancer mortality, gastric cancer incidence or mortality, and colorectal cancer incidence. Details of a priori and a posteriori DPs are shown in Tables 1 and 2, and reconstructed JDI-9 component profiles for a posteriori DPs are provided in Supplemental Table 3.

**FIGURE 1:**
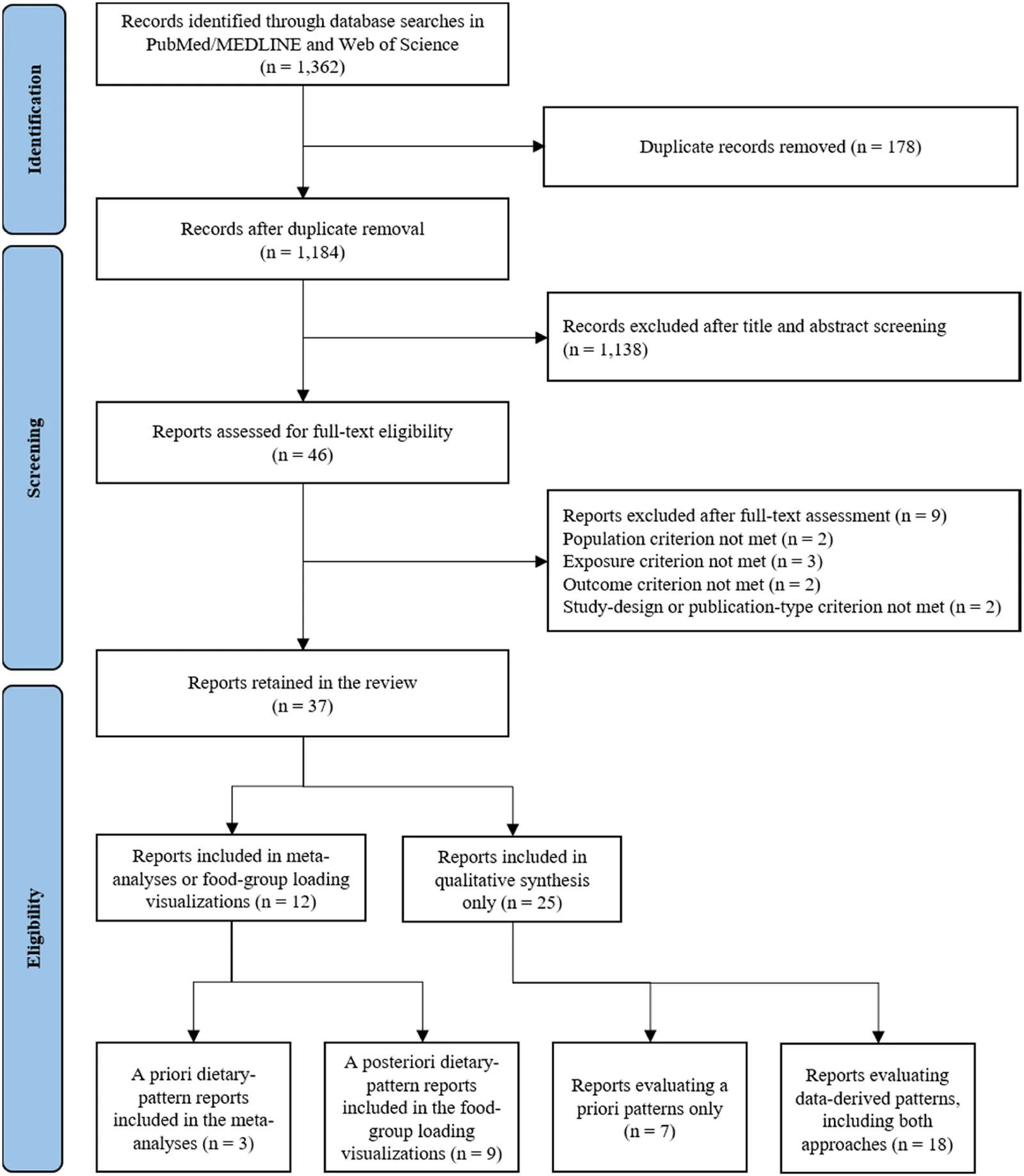
PRISMA flow diagram of study selection. The diagram summarizes identification, duplicate removal, title and abstract screening, full-text assessment, and the allocation of retained reports to the synthesis streams. Of 46 full-text reports assessed, 9 were excluded and 37 were retained, including the additional descriptive evidence specified in Supplemental Table 4. Three a priori reports contributed to meta-analyses, 9 a posteriori reports contributed to food-group loading visualizations, and 25 reports were included in qualitative synthesis only. Among the latter, 7 evaluated a priori patterns only, 15 evaluated data-derived patterns only, and 3 evaluated both approaches. The 3 dual-approach reports are counted once in the data-derived branch in this figure and are listed in Supplemental Table 6A. PRISMA, Preferred Reporting Items for Systematic Reviews and Meta-Analyses.

**TABLE 1:** Characteristics of studies included in the main meta-analyses of a priori DPs and mortality outcomes

Author, Year	Cohort/study	No. of participants	Age	Sex	Dietary assessment	Exposure	Positive scoring items	Negative scoring items	Study-specific score range and categories compared	Outcomes (used in this study)	Follow-up period	Adjusted factors	Funding
Okada 2018 [37]	JACC Study	58,767	40–79 y	Both sexes	39-item FFQ	Japanese Food Score	Beans and bean products; fresh fish; vegetables; Japanese pickles; fungi; seaweeds; fruits. One point was assigned for each food group consumed ≥3–4 times/wk	None	0–7 points; highest, 6–7 vs. lowest, 0–2 (reference); sex-specific estimates	All-cause, CVD, and cancer mortality	Median 18.9 y in men and 19.4 y in women	Age, geographic region, BMI, education duration, smoking status, alcohol drinking status, sports habits, sleep duration, history of hypertension, history of diabetes, and total energy intake	Grants-in-Aid for Scientific Research from the Ministry of Education, Culture, Sports, Science and Technology of Japan (MEXT) (Monbusho); Grants-in-Aid for Scientific Research on Priority Areas of Cancer; and Grants-in-Aid for Scientific Research on Priority Areas of Cancer Epidemiology from MEXT (MonbuKagaku-sho) (nos. 61010076, 62010074, 63010074, 1010068, 2151065, 3151064, 4151063, 5151069, 6279102, 11181101, 17015022, 18014011, 20014026, and 20390156)
Abe 2020 [38]	Ohsaki Cohort 1994 Study	14,764	40–79 y	Both sexes	40-item FFQ	Japanese Diet Index score, JDI	Rice; miso soup; seaweeds; pickles; green and yellow vegetables; fish; green tea. One point was assigned if intake was ≥ the sex-specific median	Beef and pork; coffee. One point was assigned if intake was < the sex-specific median	0–9 points; highest, Q4 (7–9) vs. lowest, Q1 (0–4; reference); combined-sex estimate	All-cause mortality, CVD mortality, cancer mortality	20 y	Cox models used age as the time scale. The fully adjusted model included sex, education level, smoking status, alcohol consumption, walking time, history of hypertension, diabetes, stroke, myocardial infarction, or cancer, BMI, and energy intake	NARO Bio-oriented Technology Research Advancement Institution (advanced integration research for agriculture and interdisciplinary fields) [grant number 17943029]
Murai 2025 [39]	JPHC Study	93,049	45–74 y	Both sexes	147-item FFQ	Traditional Japanese Diet Index score, T-JDIs	Rice; miso soup; seaweeds; pickles; vegetables other than pickles; non-salty seafood; salty seafood; green tea; fruits; soy products; mushrooms; salt by seasoning. One point was assigned if intake was ≥ the sex-specific energy-adjusted median, except green tea, seaweeds, and mushrooms, which were not energy-adjusted	Beef and pork. One point was assigned if intake was < the sex-specific energy-adjusted median	0–13 points; highest, Q4 (9–13) vs. lowest, Q1 (0–4; reference); sex-specific estimates	All-cause mortality and cause-specific mortality, including cancer, and CVD mortality	Median 18.9 y	Model 2 was stratified by public health center area and adjusted for age, BMI, smoking status, alcohol consumption, total physical activity, occupation, history of diabetes mellitus, treatment of hypertension, treatment of hypercholesterolemia, screening examination, total energy intake, and menopausal status in women	National Cancer Center Research and Development Fund (23-A-31 [toku], 26-A-2, 29-A- 4, 2020-J-4, and 2023-J-4), a Grant-in-Aid for Cancer Research from the Ministry of Health, Labour and Welfare in Japan (from 1989 to 2010), and a Grant-in-Aid for Early-Career Scientists (21K17300)

This table summarizes studies contributing to the main meta-analyses of a priori Japanese-style DPs and mortality outcomes. Score ranges and category boundaries were taken directly from the original studies. Okada et al. [37] and Murai et al. [39] contributed sex-specific estimates, whereas Abe et al. [38] contributed 1 combined-sex estimate; together, the 3 studies contributed 5 analytic estimates per outcome.

Abbreviations: DP, dietary pattern; FFQ, food frequency questionnaire; HR, hazard ratio; JACC, Japan Collaborative Cohort Study; JDI, Japanese Diet Index; JPHC, Japan Public Health Center-Based Prospective Study.

**TABLE 2:** Characteristics of studies included in the outcome-specific food-group loading visualizations of a posteriori dietary patterns

Author, Year	Cohort/study	No. of participants	Age	Sex	Dietary assessment	Dietary pattern evaluation	Outcomes (used in this study)	Follow-up period	Adjusted factors	Funding
Masaki 2003 [40]	JACC Study	5644	40–69 y	Men	33-item FFQ (with 26 items used in PCA)	PCA	Gastric cancer incidence	10 y	Age, BMI, education, history of peptic ulcer, family history of stomach cancer, status of cigarette smoking, and alcohol drinking	Grant-in-Aid for Scientific Research from the Ministry of Education, Science, Sports and Culture of Japan: 61010076, 62010074, 63010074, 1010068, 2151065, 3151064, 4151063, 5151069, 6279102
Kim 2004 [41]	JPHC Study	42,112	40–59 y	Both sexes	44-item FFQ	FA	Gastric cancer incidence	10 y	Age, BMI, energy intake, education level, and family history of gastric cancer; in men only, smoking status and alcohol drinking	Grant-in-Aid for Cancer Research and for the Second-Term Comprehensive Ten-Year Strategy for Cancer Control, Ministry of Health and Welfare of Japan and Foundation for Promotion of Cancer Research in Japan Visiting Scientist Fellowship from the Foundation for Promotion of Cancer Research in Japan
Kim 2005 [42]	JPHC Study	42,112	40–59 y	Both sexes	44-item FFQ	FA	Colorectal cancer incidence	10 y	Age, BMI, study area, energy intake, education level, physical activity, and family history of colorectal cancer; in men only, smoking status and alcohol drinking	Grant-in-Aid for Cancer Research and for the Second-Term Comprehensive Ten-Year Strategy for Cancer Control, Ministry of Health and Welfare of Japan and Foundation for Promotion of Cancer Research in Japan Visiting Scientist Fellowship from the Foundation for Promotion of Cancer Research in Japan
Shimazu 2007 [43]	Ohsaki National Health Insurance cohort study	40,547	40–79 y	Both sexes	40-item FFQ	FA	Cardiovascular disease mortality	7 y	Age, sex, smoking status, walking duration, education, total energy intake, BMI, and history of hypertension	Health Sciences Research Grant for Health Services (H18-Choju-Ippan-014, H16-Seisaku-Ippan- 023, H18-Junkankitou (Seisyu)-Ippan-012), Ministry of Health, Labour and Welfare, Japan
Pham 2010 [44]	JACC Study	63,403	40–79 y	Both sexes	38-item FFQ	FA	Gastric cancer mortality	Men: average 12.7 y Women: average 13.5 y	Age, tobacco smoking status, history of gastric ulcer, stomach cancer screening, BMI, educational level, and total energy intake	Grant-in-aid for Scientific Research on Priority Areas (2) [No. 12218237] from the Ministry of Education, Culture, Sports, Science, and Technology of Japan. The JACC Study was also supported by Grants-in-Aid for Scientific Research from the Ministry of Education, Science, Sports and Culture of Japan (Monbusho) [Nos. 61010076, 62010074, 63010074, 1010068, 2151065, 3151064, 4151063, 5151069, 6279102, 11181101, 17015022, 18014011, and 20014026]
Maruyama 2013 [45]	JACC Study	64,037	40–79 y	Both sexes	40-item FFQ	FA	Mortality from stroke, coronary artery disease, and total cardiovascular disease	Average 12.6 y	Age, BMI, smoking category, walking time, hours of sports, education, perceived mental stress, sleep duration, total energy intake, and history of hypertension and diabetes	Grants-in-Aid for Scientific Research (Nos. 61010076, 62010074, 63010074, 1010068, 2151065, 3151064, 4151063, 5151069, 6279102, 11181101, 17015022, 18014011, and 20014026) from the Ministry of Education, Culture, Sports, Science and Technology (MEXT), Japan
Kumagai 2014 [46]	Ohsaki National Health Insurance cohort study	44,097	40–79 y	Both sexes	40-item FFQ	FA	Colorectal, colon, and rectal cancer incidence	11 y	Age, sex, BMI, smoking status, walking duration, education, total energy intake, and family history of colorectal cancer	National Cancer Center Research and Development Fund (24-A-3) and the Third Term Comprehensive Ten-Year Strategy for Cancer Control, Ministry of Health, Labour and Welfare, Japan (H23-Jun- kankitou (Seisyu)-Ippan-005)
Nanri 2017 [47]	JPHC Study	81,720	45–74 y	Both sexes	147-item FFQ (134 food and beverage items retained and aggregated into 48 food groups for PCA)	PCA	All-cause and cardiovascular disease mortality	Average 14.8 y	Age, sex, study area, BMI, smoking status, total physical activity, history of diabetes mellitus, history of hypertension, and total energy intake	National Cancer Centre Research and Development Fund (23-A-31[toku] and 26-A-2) (since 2011), a Grant-in-Aid for Cancer Research from the Ministry of Health, Labour and Welfare of Japan (from 1989 to 2010), Practical Research Project for Lifestyle related Diseases including Cardiovascular Diseases and Diabetes Mellitus (15ek0210021h0002) from the Japan Agency for Medical Research and Development, JSPS KAKENHI Grant No. 15H04779, and grants-in-aid for research from the National Centre for Global Health and Medicine (26A-201)
Shin 2017 [48]	JPHC Study	93,062	45–74 y	Both sexes	138-item FFQ	PCA	Colorectal, colon, proximal colon, distal colon, and rectal cancer incidence	Average 13.8 y	Age, public health center area, BMI, smoking status, total physical activity, menopause status, log-transformed total energy intake, and use of exogenous female hormones	National Cancer Center Research and Development Fund (23-A-31 [toku] and 26-A-2) (since 2011) and a Grant-in-Aid for Cancer Research from the Ministry of Health, Labour and Welfare of Japan (from 1989 to 2010) The author was supported by the Basic Science Research Program through the National Research Foundation of Korea (NRF) funded by the Ministry of Education, Science and Technology (2014R1A6A3A03056928)

This table summarizes the studies contributing to the outcome-specific food-group loading visualizations of a posteriori dietary patterns. Abbreviations: FA, factor analysis; FFQ, food frequency questionnaire; JACC, Japan Collaborative Cohort Study; JPHC, Japan Public Health Center-Based Prospective Study; PCA, principal component analysis.

### A priori Japanese-style DPs and mortality outcomes

Meta-analyses of a priori Japanese-style DPs provided the main quantitative evidence (Table 3). Pooled estimates comparing the highest and lowest score categories suggested lower all-cause and CVD mortality. For all-cause mortality, the pooled HR was 0.88 (95% CI: 0.83, 0.93; *P* < 0.001; 3 studies; 5 estimates), with substantial heterogeneity (*I*^2^ = 61.1%; *P* for heterogeneity = 0.04). For CVD mortality, the pooled HR was 0.82 (95% CI: 0.73, 0.93; *P* = 0.002; 3 studies; 5 estimates), with substantial heterogeneity (*I*^2^ = 71.6%; *P* for heterogeneity = 0.01). The pooled estimate for total cancer mortality was close to the null (HR: 1.01; 95% CI: 0.95, 1.06; *P* = 0.85; 3 studies; 5 estimates; *I*^2^ = 0.0%; *P* for heterogeneity = 0.66). The corresponding forest plots are shown in Figure 2.

**TABLE 3:** Meta-analysis of a priori Japanese-style DPs and mortality outcomes

Outcomes	Highest vs. lowest							
	No. of studies	*n*	Event (*n*)	HR	95% CI	*P* value	*I*^2^ (%)	*P* for heterogeneity
All-cause mortality	3	166,580	39,649	0.88	0.83–0.93	<0.001	61.1	0.04
CVD mortality	3	166,580	10,792	0.82	0.73–0.93	0.002	71.6	0.01
Total cancer mortality	3	166,580	13,838	1.01	0.95–1.06	0.85	0.0	0.66

Estimates compare the highest and lowest score categories. Sex-specific estimates were treated as separate analytic strata only when an overall estimate was not reported.

Abbreviations: CI, confidence interval; CVD, cardiovascular disease; HR, hazard ratio.

**FIGURE 2:**
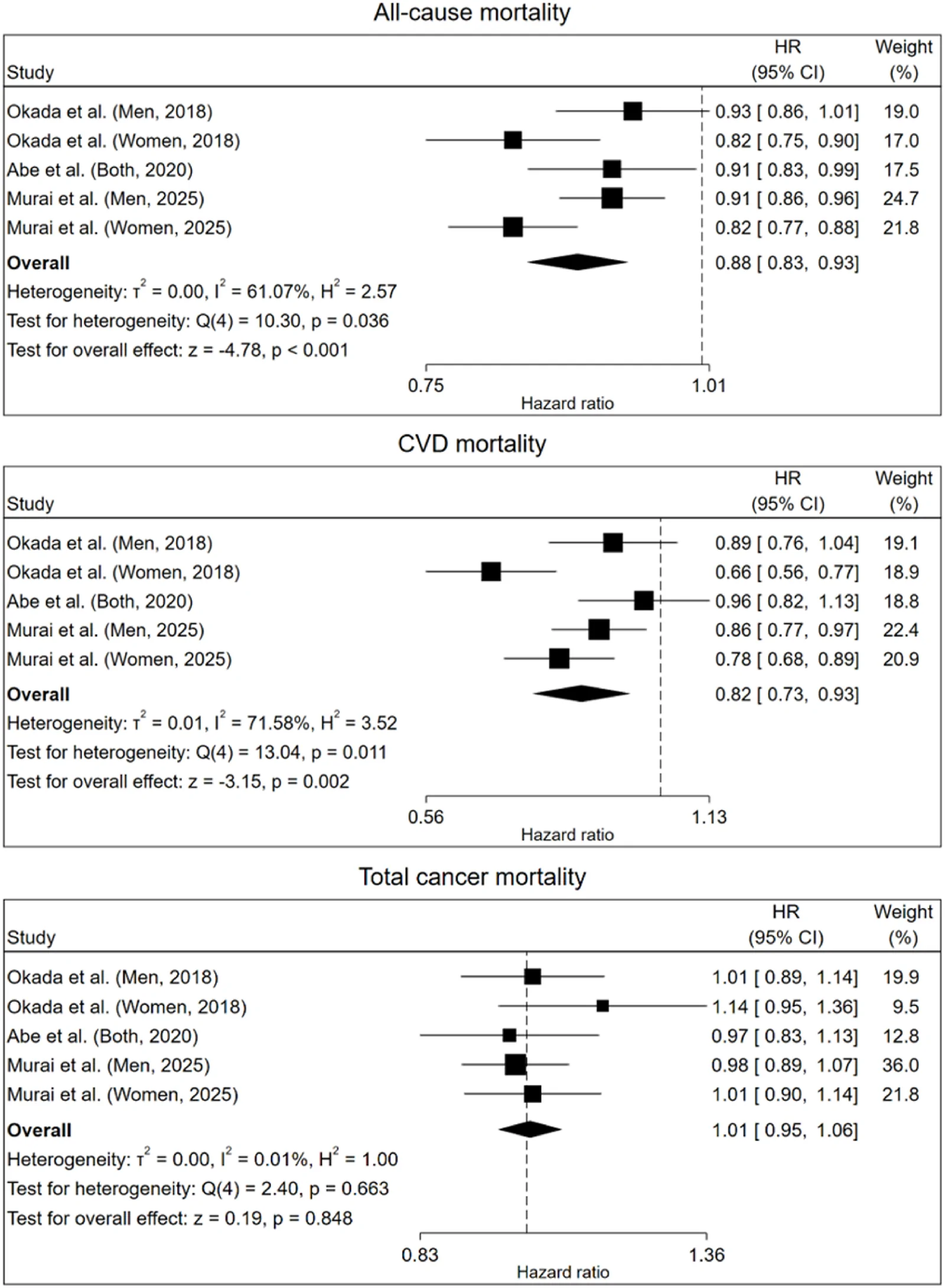
Random-effects meta-analysis of a priori Japanese-style dietary patterns and mortality outcomes. From top to bottom, the forest plots show all-cause mortality, cardiovascular disease mortality, and total cancer mortality. Hazard ratios compare the highest with the lowest score categories. Pooled estimates were generated using restricted maximum-likelihood random-effects models. Meta-analyses were conducted only for a priori Japanese-style dietary patterns. CI, confidence interval; CVD, cardiovascular disease; HR, hazard ratio; REML, restricted maximum likelihood.

### JDI-9 similarity and pattern names of a posteriori DPs

Among the 43 a posteriori DPs from 9 studies included in the auxiliary food-group loading visualizations, 21 were classified as JDI-9-like and 22 as non–JDI-9-like under the primary conjunctive rule. This classification was based on the reconstructed JDI-9 component vectors rather than on investigator-assigned DP names. Supplemental Table 3 presents, for each DP, the reconstructed loadings for the 9 JDI-9 components, the number of available components, cosine similarity score, distance score, metric-specific quartile categories, and final classification. The 2 metric-specific ordinal classifications were correlated (Spearman’s ρ = 0.90; *P* < 0.001). The 2 metrics agreed on whether the Closest or Close criterion was met for 41 of the 43 DPs (95.3%): 21 DPs met the criterion for both metrics, 20 met it for neither metric, and 2 were metric-discordant. In the single-metric sensitivity check, cosine-only and distance-only labeling each classified 22 DPs as JDI-9-like. The “Vegetable and fruit” pattern reported by Masaki et al. [40] met only the cosine-similarity criterion, whereas the women’s “Animal food” pattern reported by Maruyama et al. [45] met only the distance-score criterion. Investigator-assigned pattern names among the DPs classified as JDI-9-like under the primary rule included “Japanese,” “traditional,” “healthy,” “prudent,” “vegetable,” and “animal food.”

### Food-group loading visualization of a posteriori DPs and selected outcomes

The outcome-specific food-group loading visualizations are shown in FIGURE 3, FIGURE 4, FIGURE 5, FIGURE 6. Each figure presents, for each a posteriori DP, the reported effect estimate and 95% CI, investigator-assigned DP label, JDI-9-like classification, and food-group loadings. For all-cause mortality, the “Prudent” and “Traditional Japanese” DPs were classified as JDI-9-like, but their reported estimates differed; a non–JDI-9-like “Westernized” DP had a point estimate in the lower-risk direction. For CVD mortality, JDI-9-like DPs labeled “Prudent,” “Japanese,” or “Vegetable” had point estimates in the lower-risk direction. Across these patterns, positive loadings were reported for vegetables, seaweed, fish, fruits, legumes, mushrooms, and potatoes. Point estimates in the lower-risk direction were also reported for non–JDI-9-like DPs, including “Dairy product” and “Westernized” patterns. For gastric cancer incidence, the JDI-9-like “Healthy” and “Traditional” DPs had estimates in different directions. The “Traditional” DP had higher loadings for rice, miso or miso soup, fish, and pickles, whereas the “Healthy” DP had positive loadings across a wider range of plant-based and contemporary food groups. For gastric cancer mortality, the reported estimates varied across the displayed a posteriori DPs. For colorectal cancer incidence, the displayed DPs differed in loadings for vegetables, seaweed, fruits, dairy products, legumes, mushrooms, meat, coffee, and alcohol.

**FIGURE 3:**
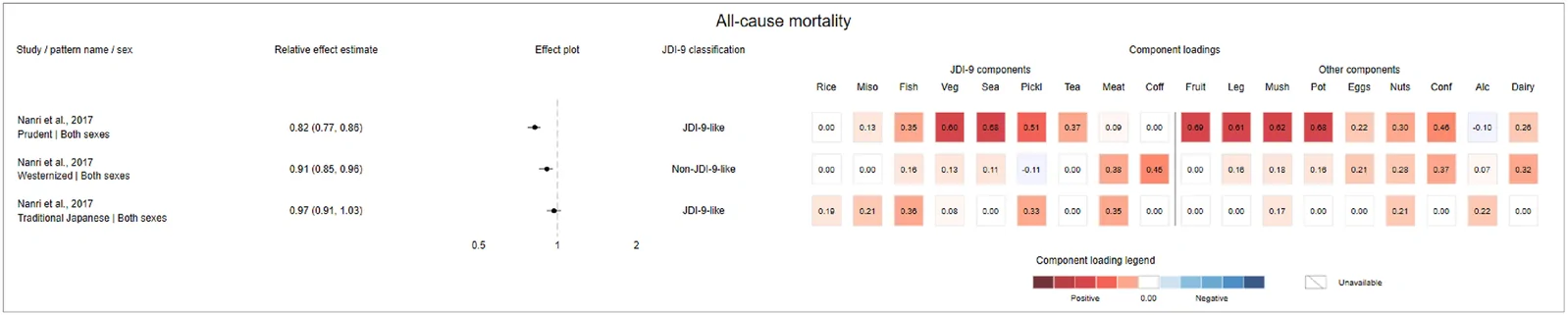
Food-group loading visualization of a posteriori dietary patterns and all-cause mortality. The figure displays reported relative effect estimates, 95% CIs, investigator-assigned pattern names, sex strata where applicable, JDI-9-like classification, and reconstructed food-group loadings for longitudinal a posteriori dietary patterns reporting all-cause mortality. The visualization displays component-level food structures alongside reported associations. Food-group loadings are shown as a heatmap: positive loadings are displayed as red intensity gradients, negative loadings as blue intensity gradients, zero loadings as white cells, and unavailable food groups as white cells with diagonal hatching. Food-group abbreviations: Rice, rice; Miso, miso or miso soup; Fish, fish and shellfish; Veg, vegetables; Sea, seaweed; Pickl, pickles; Tea, green tea; Meat, meat; Coff, coffee; Fruit, fruit; Leg, legumes; Mush, mushrooms; Pot, potatoes; Eggs, eggs; Nuts, nuts; Conf, confectionery products; Alc, alcohol; Dairy, dairy products. JDI-9-like classification was based on reconstructed loadings for the 9 JDI-9 reference components: rice, miso or miso soup, fish and shellfish, vegetables, seaweed, pickles, green tea, meat, and coffee. CI, confidence interval; DP, dietary pattern; HR, hazard ratio; JDI-9, Japanese Diet Index-9.

**FIGURE 4:**
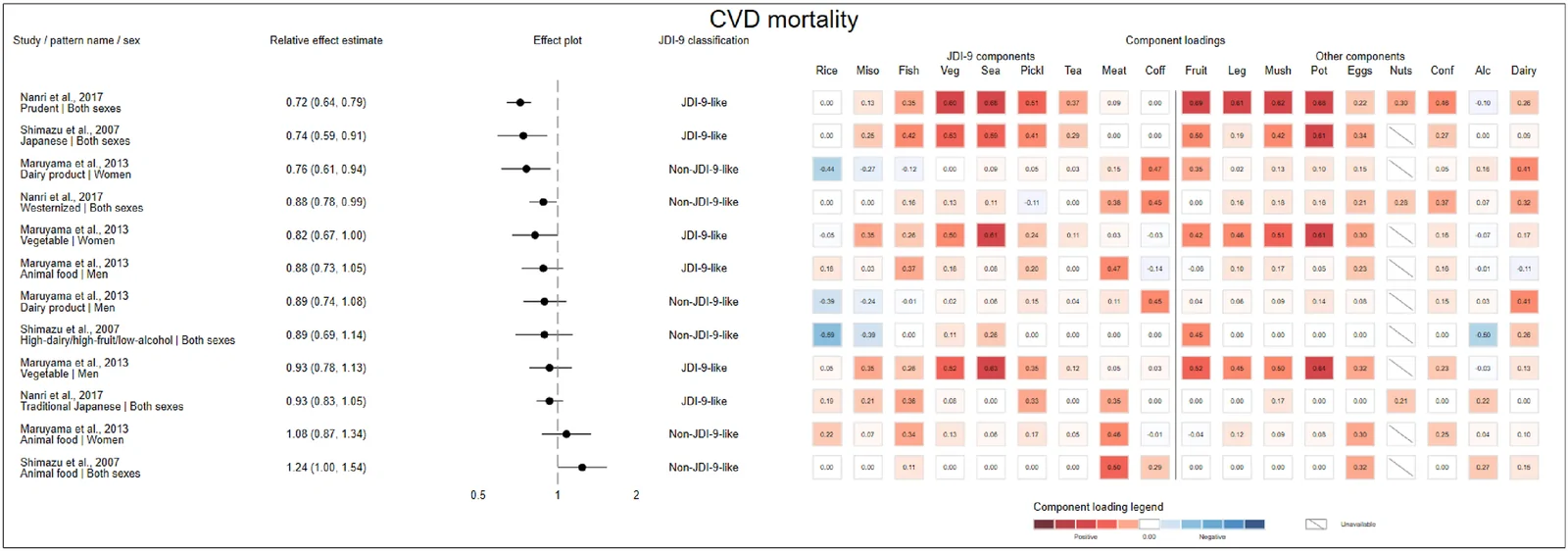
Food-group loading visualization of a posteriori dietary patterns and cardiovascular disease mortality. The figure displays reported relative effect estimates, 95% CIs, investigator-assigned pattern names, sex strata where applicable, JDI-9-like classification, and reconstructed food-group loadings for longitudinal a posteriori dietary patterns reporting cardiovascular disease mortality. The visualization displays food-structural features alongside reported associations. Food-group loadings are shown as a heatmap: positive loadings are displayed as red intensity gradients, negative loadings as blue intensity gradients, zero loadings as white cells, and unavailable food groups as white cells with diagonal hatching. Food-group abbreviations: Rice, rice; Miso, miso or miso soup; Fish, fish and shellfish; Veg, vegetables; Sea, seaweed; Pickl, pickles; Tea, green tea; Meat, meat; Coff, coffee; Fruit, fruit; Leg, legumes; Mush, mushrooms; Pot, potatoes; Eggs, eggs; Nuts, nuts; Conf, confectionery products; Alc, alcohol; Dairy, dairy products. JDI-9-like classification was based on reconstructed loadings for the 9 JDI-9 reference components: rice, miso or miso soup, fish and shellfish, vegetables, seaweed, pickles, green tea, meat, and coffee. CI, confidence interval; CVD, cardiovascular disease; DP, dietary pattern; HR, hazard ratio; JDI-9, Japanese Diet Index-9.

**FIGURE 5:**
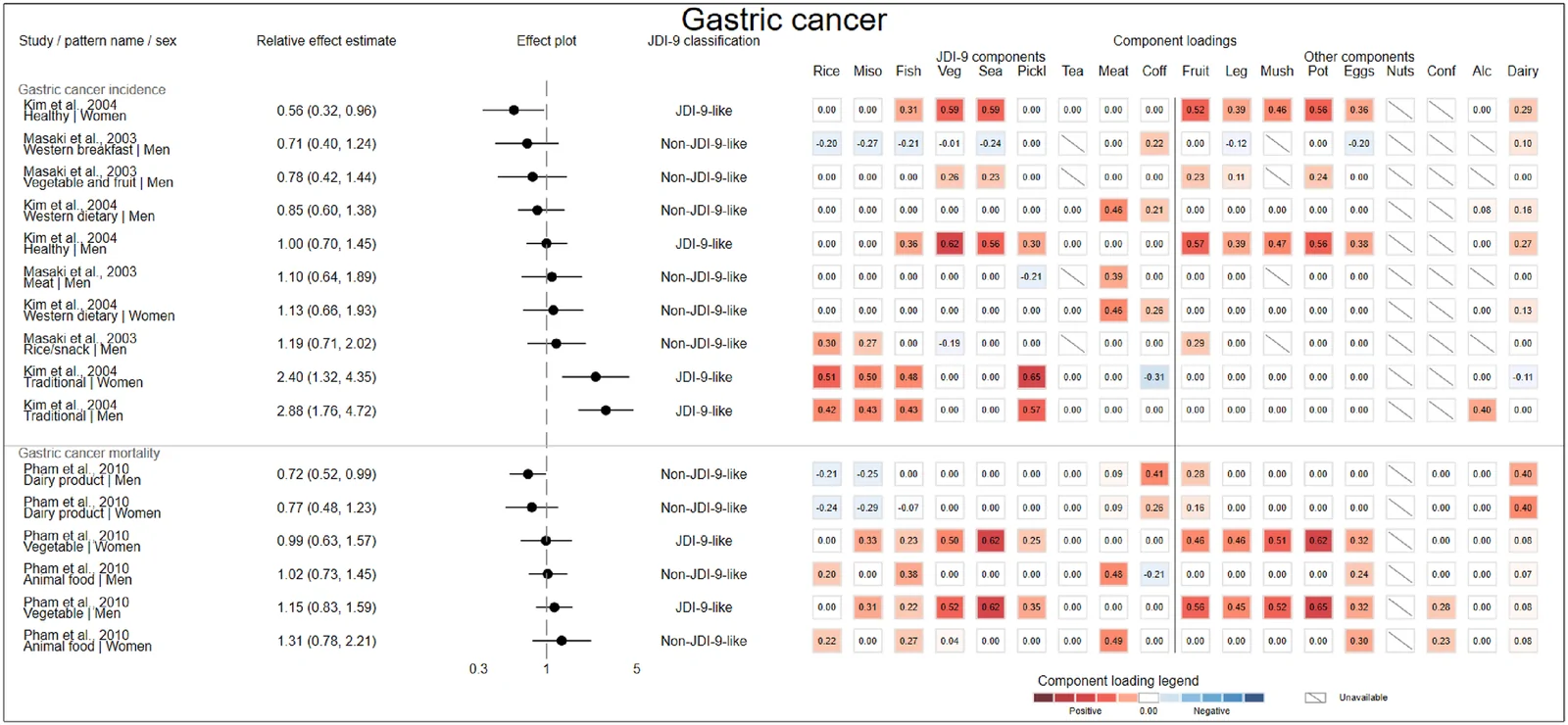
Food-group loading visualization of a posteriori dietary patterns and gastric cancer incidence or mortality. The figure displays reported relative effect estimates, 95% CIs, investigator-assigned pattern names, sex strata where applicable, JDI-9-like classification, and reconstructed food-group loadings for longitudinal a posteriori dietary patterns reporting gastric cancer incidence or gastric cancer mortality. The upper group shows incidence and the lower group shows mortality. The visualization displays differences in component-level food structures among patterns with similar Japanese-style features, including loadings for plant-based foods and high-sodium traditional components such as miso or miso soup and pickles. Food-group loadings are shown as a heatmap: positive loadings are displayed as red intensity gradients, negative loadings as blue intensity gradients, zero loadings as white cells, and unavailable food groups as white cells with diagonal hatching. Food-group abbreviations: Rice, rice; Miso, miso or miso soup; Fish, fish and shellfish; Veg, vegetables; Sea, seaweed; Pickl, pickles; Tea, green tea; Meat, meat; Coff, coffee; Fruit, fruit; Leg, legumes; Mush, mushrooms; Pot, potatoes; Eggs, eggs; Nuts, nuts; Conf, confectionery products; Alc, alcohol; Dairy, dairy products. JDI-9-like classification was based on reconstructed loadings for the 9 JDI-9 reference components: rice, miso or miso soup, fish and shellfish, vegetables, seaweed, pickles, green tea, meat, and coffee. CI, confidence interval; DP, dietary pattern; HR, hazard ratio; JDI-9, Japanese Diet Index-9.

**FIGURE 6:**
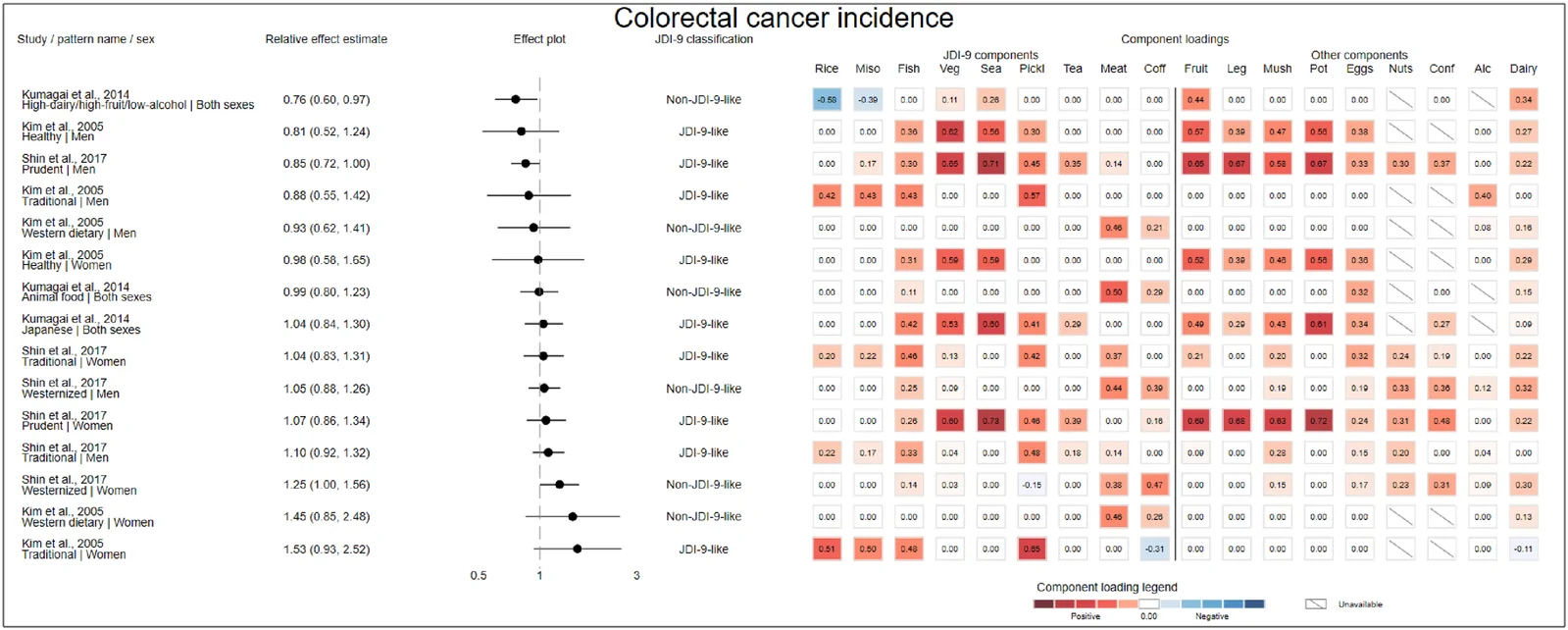
Food-group loading visualization of a posteriori dietary patterns and colorectal cancer incidence. The figure displays reported relative effect estimates, 95% CIs, investigator-assigned pattern names, sex strata where applicable, JDI-9-like classification, and reconstructed food-group loadings for longitudinal a posteriori dietary patterns reporting colorectal cancer incidence. The visualization compares investigator-assigned pattern names, JDI-9-like classification, and component-level food structures in relation to reported estimates. Food-group loadings are shown as a heatmap: positive loadings are displayed as red intensity gradients, negative loadings as blue intensity gradients, zero loadings as white cells, and unavailable food groups as white cells with diagonal hatching. Food-group abbreviations: Rice, rice; Miso, miso or miso soup; Fish, fish and shellfish; Veg, vegetables; Sea, seaweed; Pickl, pickles; Tea, green tea; Meat, meat; Coff, coffee; Fruit, fruit; Leg, legumes; Mush, mushrooms; Pot, potatoes; Eggs, eggs; Nuts, nuts; Conf, confectionery products; Alc, alcohol; Dairy, dairy products. JDI-9-like classification was based on reconstructed loadings for the 9 JDI-9 reference components: rice, miso or miso soup, fish and shellfish, vegetables, seaweed, pickles, green tea, meat, and coffee. CI, confidence interval; DP, dietary pattern; HR, hazard ratio; JDI-9, Japanese Diet Index-9.

### Qualitative synthesis

Twenty-five reports were synthesized qualitatively: 17 longitudinal and 8 cross-sectional. Seven evaluated a priori patterns only, 15 data-derived patterns only, and 3 both approaches. Their characteristics and findings are presented in Supplemental Table 6.

Mortality findings included inverse associations for Japanese-style and plant-food patterns, but cancer-mortality intervals included the null and some all-cause mortality comparisons were inconclusive (Supplemental Table 6; Supplemental References [1], [6], [11], and 23).

Several longitudinal reports showed inverse associations with functional disability or dementia, although exposure definitions and score-change contrasts differed. Fracture estimates were imprecise, and frailty associations varied by pattern. Cross-sectional associations with sarcopenia and cognitive outcomes differed by sex, dietary score, and outcome, with some comparisons showing no clear association (Supplemental Table 6; [2], [3], [4], [5], [7], [8], [9], [13], [14], [15], 19, 24, and 25).

Other cancer findings varied by pattern and subgroup, including higher breast or prostate cancer risk for Westernized patterns and inverse associations for selected patterns (Supplemental Table 6; Supplemental References [17], [18], [20] and 22).

For cardiometabolic measures, a longitudinal report associated higher Japanese-pattern scores with smaller increases in selected blood pressure measures. Cross-sectional findings varied by pattern, sex, and outcome, with inverse, positive, and unclear associations (Supplemental Table 6; [10], [12], [16] and 21).

### Risk of bias

Among the 12 studies included in the analytic syntheses, the overall ROBINS-E judgments were “some concerns” for 9 studies and “high risk of bias” for 3 studies: Abe et al. [38], Masaki et al. [40], and Pham et al. [44]. Domain 4 was judged to be at low risk of bias in all 12 studies because, based on the information reported, no relevant postexposure intervention that was influenced by prior dietary exposure and could affect the outcome was identified. The absence of repeated dietary measurements and possible changes in habitual diet during follow-up were considered under Domain 2.

Specifically, Abe et al. [38] was judged to be at high risk of bias in Domain 5 (bias because of missing data) because >50% of eligible participants (14,922 of 29,686) were excluded from the primary complete-case analysis owing to missing dietary data, which could have biased the reported effect estimates. Masaki et al. [40] was judged to be at high risk of bias in both Domain 1 (bias because of confounding) and Domain 2 (bias arising from measurement of the exposure). The Domain 1 judgment reflected the lack of adjustment for total energy intake, which had been prespecified as an important confounder in DP analyses. The Domain 2 judgment reflected the use of a single dietary assessment without portion-size information, the possibility that data collected during summer did not represent usual annual intake, and the lack of established validation for the derived DP measures. Pham et al. [44] was judged to be at high risk of bias in Domain 1 because physical activity, which had been prespecified as an important potential confounder of the DP–outcome association, was not adjusted for in the multivariable analysis. Detailed domain-level and overall judgments are provided in Supplemental Table 2.

Among the 17 longitudinal reports included in the qualitative synthesis only, 10 were judged at high risk of bias and 7 had some concerns. The 8 cross-sectional reports received domain-level assessments without overall judgments. High-risk judgments in the additional reports involved confounding, exposure measurement, missing data, or selection of reported results; the domain-level judgments and result-specific distinctions are presented in Supplemental Table 7.

## Discussion

### Main findings

Pooled estimates from 3 independent studies suggested lower all-cause and CVD mortality with greater alignment with a priori Japanese-style DPs, although heterogeneity was substantial for both outcomes; the pooled estimate for total cancer mortality was close to the null. Food-group loading visualization of a posteriori DPs illustrated component-level variation that was not apparent from investigator-assigned pattern names or binary JDI-9-like classification alone. Together, these observations informed a provisional framework describing Japanese-style DPs as food-structural profiles that include potentially favorable traditional components, high-sodium traditional components, and contemporary dietary components.

### A priori Japanese-style dietary patterns and health outcomes

The lower pooled estimates for all-cause and CVD mortality were consistent with the CVD-focused meta-analysis by Shirota et al. [6]. Although based on limited longitudinal evidence, they suggest that predefined Japanese-style scores may capture mortality-related dietary characteristics in the included cohorts.

Japanese-style DPs commonly include fish, soy products, vegetables, seaweed, green tea, and other plant-based foods. In Japan, green tea is generally consumed without added sugar and may provide tea polyphenols, including catechins. Its unsweetened form helps distinguish this component from sweetened tea beverages in some non-Japanese dietary contexts [49], [50]. These components may contribute to higher intakes of dietary fiber, potassium, folate, vitamin C, marine n–3 fatty acids, plant-derived protein, and other nutrients discussed in relation to cardiometabolic health and mortality outcomes [51], [52], [53], [54], [55], [56], [57], [58], [59], [60], [61]. However, these components coexist with sodium-rich foods and seasonings, with potentially different implications for blood pressure, stroke, and gastric cancer [62], [63], [64], [65].

The pooled estimate for total cancer mortality was close to the null, but this outcome combines cancer sites with distinct etiologies and dietary determinants. In Japan, infection-related factors, smoking, and alcohol account for substantial population-attributable fractions, whereas diet- and adiposity-related factors account for smaller fractions [66]. A summary estimate may therefore obscure site-specific associations, including those of sodium-rich foods with gastric cancer and of fruits, dietary fiber, alcohol, meat, dairy products, and calcium with colorectal cancer [67], [68], [69], [70], [71]. Cancer associations should therefore be evaluated by site and food structure [72].

These results inform the interpretation of a priori Japanese-style DPs. A priori Japanese-style DPs are useful because they provide explicit, reproducible scoring frameworks for examining Japanese-style food structures across studies. At the same time, many existing scores rely on study-specific medians or quantiles rather than absolute intake criteria. Therefore, a person assigned to a high-score category in one cohort may not have the same absolute intake profile as a person assigned to a high-score category in another cohort. This limitation is important when moving from within-cohort epidemiologic analysis toward cross-study comparison and future health outcome–based definitions of Japanese-style DPs.

### A posteriori dietary patterns and refinement of Japanese-style DP research

The joint display of effect estimates, investigator-assigned names, JDI-9 similarity, and food-group loadings enabled comparison of a posteriori DPs at the level of food structure and revealed heterogeneity within similarly named and JDI-9-like patterns.

The all-cause mortality visualization illustrates this heterogeneity: the JDI-9-like “Prudent” and “Traditional Japanese” patterns differed in their reported associations and loading breadth, whereas a non–JDI-9-like “Westernized” pattern showed a point estimate in the lower-risk direction. This contrast suggests that non–JDI-9-like DPs may capture contemporary food combinations or dietary variety rather than uniformly unfavorable diets [29], [32], [73], [74]. For CVD mortality, point estimates in the lower-risk direction occurred in JDI-9-like plant- and fish-rich patterns and in some non–JDI-9-like “Dairy product” or “Westernized” patterns. Together, the loadings reflected contrasts among plant- and fish-rich structures, staple food dominance, beverage patterns, and contemporary food combinations rather than isolated effects of individual foods.

Gastric cancer illustrates why sodium-sensitive interpretation is important, particularly in Japan, where gastric cancer has historically been a major public health burden and salt-preserved foods have long been implicated [75], [76]. In Kim et al., patterns labeled “Healthy” and “Traditional” were both JDI-9-like but showed associations in different directions; the former had broader plant-based and dairy loadings, whereas the latter loaded more strongly on rice, miso or miso soup, fish, and pickles. Thus, traditional Japanese-style similarity and a health-oriented food structure may not be equivalent for sodium-sensitive outcomes [41].

The colorectal cancer visualization likewise showed that pattern names and JDI-9-like classification did not consistently distinguish associations. In Japan, where colorectal cancer has become a leading cancer site, a non–JDI-9-like pattern characterized by dairy products, fruit, and low alcohol intake showed a point estimate in the lower-risk direction, whereas several JDI-9-like patterns were compatible with the null. This contrast is compatible with evidence that dairy products and calcium may be relevant to colorectal cancer risk [77]. Given the contribution of broader lifestyle factors, these findings remain descriptive and support component-level rather than label-based interpretation.

### Toward a health-oriented Japanese-style dietary pattern

These findings support developing and validating health-oriented Japanese-style DPs against specific outcomes [78], [79]. On the basis of the present synthesis, we propose a provisional 3-layer framework [80]: potentially favorable traditional components, such as fish, soy products, vegetables, seaweed, and unsweetened green tea; high-sodium traditional components, such as miso soup, pickles, salted seafood, and seasoning-derived salt; and contemporary components not consistently included in existing Japanese diet indices, such as fruits, dairy products, coffee, legumes, mushrooms, potatoes, eggs, and nuts, with alcohol, confectioneries, and meat requiring outcome-specific interpretation. This framework links historical data on the composition of Japanese-style DPs with evidence on health outcomes. It may inform the evaluation of Japanese-style DPs and the development of reproducible, health outcome–oriented dietary scores.

Several implications follow from this framework. First, future Japanese-style DP research should move beyond relative ranking alone. Many a priori Japanese-style DPs, including those in the JDI family, assign component scores according to medians or quantiles within each study population [31], [38], [81], [82], [83], [84], [85], [86], [87], [88]. This approach is useful for within-cohort analyses, but it limits comparability across populations because individuals assigned to high-score categories in different cohorts may not have similar absolute intake profiles. Food-group–specific criteria for potentially favorable traditional components, high-sodium traditional components, and contemporary components with potential outcome relevance should therefore be calibrated using intake distributions, feasibility, biomarkers, and health outcome evidence [89], [90].

Fermentation may also warrant consideration within broad food-group categories, particularly for soy foods and dairy products, although whether distinguishing fermented from nonfermented forms improves the outcome-specific characterization of health-oriented Japanese-style DPs requires further study [91], [92], [93]. More broadly, variation in food groups beyond the traditional core across the outcome-specific visualizations underscores the need for improved reporting of a posteriori DPs [94]. Complete food-group loadings, the rationale for DP naming, the proportion of variance explained, and the direction of major loadings should be reported transparently [95], [96], [97], [98], [99], [100], [101], [102], [103], [104], [105], [106], [107], [108], [109], [110], [111], [112]. Such reporting would improve cross-study comparison, reproducibility, and evidence synthesis. Future work should test whether health outcome–oriented Japanese-style DP frameworks can be adapted to non-Japanese populations [113], [114], [115]. Validation should focus on comparable food-structural profiles rather than literal cultural replication.

### Strengths and limitations

#### Strengths

This review distinguished between quantitatively comparable a priori evidence and descriptively synthesized a posteriori evidence. This avoided inappropriate pooling while allowing the 2 approaches to inform a common interpretation. It also updated the longitudinal evidence on mortality and developed outcome-specific visualizations displaying reported effect estimates, investigator-assigned pattern names, relative JDI-9 similarity, and food-group loadings. This component-level approach enabled differences to be identified that would not have been apparent from pattern labels or summary classifications alone, and it could be useful for synthesizing comparative evidence in other DP contexts.

#### Limitations

Several limitations should be considered. First, most included studies relied on a single self-reported dietary assessment, generally a food frequency questionnaire. Measurement error, limited food-item coverage, and changes in dietary habits during follow-up could therefore not be fully addressed. Risk-of-bias concerns also differed in their relevance to the synthesis. The study by Abe et al. [38], which was judged at high risk of bias largely because of missing dietary data, contributed to all 3 mortality meta-analyses. In contrast, the studies by Masaki et al. [40] and Pham et al. [44], which were judged at high risk because of confounding and, for Masaki et al. [40], additional exposure-measurement limitations, contributed only to the descriptive gastric cancer visualizations. For the remaining 9 studies in the analytic syntheses, some concerns persisted, but the likely direction and magnitude of any resulting bias were uncertain.

Second, the mortality meta-analyses were based on only 3 independent cohort studies, despite 5 analytic estimates being available for each outcome because some studies reported sex-specific results. Heterogeneity was substantial for all-cause mortality (*I*^2^ = 61.1%) and CVD mortality (*I*^2^ = 71.6%). Differences in score composition and scoring rules, participant characteristics, follow-up duration, and covariate adjustment may have contributed to this heterogeneity, but the number of independent studies was too small for reliable subgroup analyses or meta-regression. Accordingly, the pooled estimates should be regarded as summaries of a limited and heterogeneous evidence base rather than as precise population-average effects.

Third, the a posteriori synthesis was descriptive and depended on the information reported in the original publications. Differences in the food groups assessed and in the thresholds used to report factor loadings may have affected the reconstructed food structures. Assigning 0.00 to assessed food groups whose loadings were unreported or below the study-specific reporting threshold was a pragmatic harmonization rule and should not be interpreted as evidence of a true zero loading. In addition, the JDI-9-like classification was based on relative thresholds within the assembled set of DPs and has not been externally validated. It should therefore be interpreted as a food-structural annotation for comparative visualization rather than as a validated measure of dietary healthfulness.

Finally, the selected analytic outcomes and comparability requirements were narrower than the registered review scope. The 25 other retained reports, including cross-sectional studies, were summarized qualitatively. PubMed/MEDLINE and Web of Science Core Collection provided complementary biomedical and multidisciplinary coverage. Screening the reference lists of all 12 analytic reports identified no additional eligible reports, supporting the coverage of the mortality and selected cancer evidence retrieved through 14 March 2026. The available evidence was derived primarily from Japanese populations, and external validation is needed before the proposed framework is applied in other populations or dietary contexts.

## Conclusions

Pooled estimates from 3 independent studies suggested lower all-cause and CVD mortality with greater alignment with a priori Japanese-style DPs in Japanese populations, although the small evidence base and substantial heterogeneity limit the certainty and generalizability of these estimates. Food-group loading visualization of a posteriori DPs indicated that JDI-9-like classification alone did not consistently distinguish associations with selected health outcomes and that food-group loadings were needed for more specific interpretation. Together, these findings support the further development and outcome-specific validation of health-oriented Japanese-style DP frameworks that build on existing Japanese diet indices while distinguishing potentially favorable traditional components, high-sodium traditional components, and contemporary dietary components.

## Author contributions

The authors’ responsibilities were as follows – All authors: conceived the study and designed the study protocol; TY, MY, KO: conducted study selection and independently extracted data from the included reports; TY, MY: compiled the final analytic dataset; TY: analyzed the data, performed the statistical analyses, and drafted the manuscript; TY, MY, KO, SN, NY, ST: reviewed and edited the manuscript; SN, NY, ST: supervised the study; TY: had primary responsibility for the final content; and all authors: reviewed and approved the final manuscript.

## Data availability

The data described in the manuscript, the codebook, and the analytic code will be made available upon reasonable request, subject to approval by the corresponding author and any applicable restrictions.

## Declaration of generative AI and AI-assisted technologies in the writing process

During the preparation of this work, the authors used ChatGPT in order to support language editing and readability improvement. After using this tool, the authors reviewed and edited the content as needed and take full responsibility for the content of the publication.

## Funding

This study was supported by a joint research grant from Ajinomoto Co., Inc. and Meiji Co., Ltd. The funders had no role in the study design, data collection and analysis, interpretation of data, decision to publish, or preparation of the manuscript.

## Conflict of interest

TY is a full-time employee of Meiji Co., Ltd., and KO is a full-time employee of Ajinomoto Co., Inc. TY, MY, KO, SN, NY, and ST are affiliated with the Department of Food, Nutrition and Health, Graduate School of Public Health, International University of Health and Welfare. The department operates as a social cooperation program supported by Ajinomoto Co., Inc. and Meiji Co., Ltd. This review was supported by a joint research grant from Ajinomoto Co., Inc. and Meiji Co., Ltd. The authors declare that Ajinomoto Co., Inc. and Meiji Co., Ltd. had no role in the study design, data collection and analysis, interpretation of data, decision to publish, or preparation of the manuscript.
