# Supplementary material for: Japanese-Style Dietary Patterns, Mortality, and Selected Cancer Outcomes in Japanese Populations: A Systematic Review Integrating Meta-Analysis, Food-Group Loading Visualization, and Qualitative Synthesis
Source: Adv Nutr. 2026 Oct 15;17(12):100756. doi: 10.1016/j.advnut.2026.100756 (PMC13756781; doi:10.1016/j.advnut.2026.100756)
Supplement: Supplementary file 1 [file mmc1.docx]

**Supplementary Material**

**Supplementary Table 1. Domain-specific operational criteria used for the ROBINS-E assessment in the present review.**

| **Domain** | **Explanation** | Judgments |
| --- | --- | --- |
| 1. Risk of bias due to confounding | • Is there potential for confounding of the effect of exposure in this study?  • Did the authors use a multivariable-adjusted analysis method that controlled for the prespecified important confounding factors relevant to the exposure–outcome relation, such as age, sex where applicable, markers of body size or adiposity, smoking, physical activity, education or socioeconomic status, total energy intake, and outcome-specific factors?  • Were confounding factors that were controlled for measured validly and reliably by the variables available in this study?  • Did the authors avoid adjusting for post-exposure variables?  Notes: Confounding is expected in all observational studies; thus, no study was assigned low risk of bias. Time-varying confounding was considered unlikely to introduce material bias in the present review. | **Low risk of bias:**  No bias expected due to confounding, including time-varying confounding.  **Some concerns (moderate risk of bias):** The analysis controlled for most prespecified important confounding factors relevant to the exposure–outcome relation, but residual confounding remained possible because one or more factors were not measured or not fully controlled. Any resulting uncontrolled confounding was judged unlikely to be substantial.  Or The authors statistically investigated whether the confounding factors have an effect on the risk estimate and excluded the confounder from the multivariable model if there was no effect on the overall effect estimate. **High risk of bias:** One or more prespecified important confounding factors were not measured or appropriately controlled, and the resulting uncontrolled confounding was judged likely to be substantial. Or The authors adjusted for post-exposure variables that are affected by exposure (e.g. sodium intake and risk of stroke [adjustment for blood pressure during follow-up = intermediate biological variable on the causal pathway] -> overadjustment). **Very high risk of bias:** No adjustment was made at all for any covariate. The authors controlled for post-exposure variables, and the use of negative controls, or other considerations, suggest serious uncontrolled confounding. No information: No information is provided on which confounders were controlled for in the analysis. |
| 2. Risk of bias arising from exposure measurement | • Does the measured exposure adequately characterize the exposure metric specified to be of interest in this study?  • Was the exposure likely to be measured with error, or misclassified?  Notes: Because dietary exposure was assessed before ascertainment of subsequent outcomes, differential exposure misclassification related to outcome status was considered unlikely (Freedman et al., 2011). However, some degree of non-differential measurement error could not be excluded. The absence of repeated dietary measurements and possible changes in habitual diet during follow-up were also considered when judging whether the baseline dietary assessment adequately characterized the exposure metric and exposure period of interest. Given the general reliance on a single self-reported dietary assessment, no study was judged to be at low risk of bias in this domain. | **Low risk of bias:**  Exposure status is well-defined by the measurement and no measurement error is expected in its assessment or exposure was measured at multiple times, and exposure is stable or changes only slightly over time or exposure was measured with a single measurement, but exposure can be assumed to be stable over time. **Some concerns (moderate risk of bias):** Exposure status is well-defined by the measurement, and exposure was measured using an established or validated tool (e.g., a validated FFQ, multiple 24-h recalls) and exposure was measured at multiple times, and exposure is stable or changes only slightly over time or exposure was measured at a single time point, but exposure can be assumed to be stable over time. **High risk of bias:** Exposure status is not well characterized by the measurement (e.g., assumed from an indirect measurement) and/or exposure was measured using an unvalidated tool and/or exposure was measured with a single measurement, which is unlikely to characterize exposure during the period (e.g., single 24-h recall) or exposure cannot be assumed to be stable over time. No information: No definition of exposure or no explanation of the source of information about exposure status is reported. |
| 3. Risk of bias in selection of participants into the study | • Was selection of participants into the study (or into the analysis) based on participant characteristics observed after the start of the exposure window being studied?  • Do the start of follow-up and the start of exposure coincide for most participants?  Notes: In observational studies, it is unlikely that post-exposure variables influenced selection of participants into the study. Exclusion of participants may be mostly based on missing data, which will be considered in the domain addressing missing data (see below). The start of follow-up is considered to coincide with the baseline exposure assessment. However, participants are already exposed at the start of the study, which may have influenced outcomes measured shortly after the start of the study. | **Low risk of bias:**  All participants who would have been eligible for the target study were included in the study and start of exposure and follow-up coincide.  **Some concerns (moderate risk of bias):** Selection into the study may have been related to exposure and outcome (e.g. inclusion of postmenopausal women only); and the authors used appropriate methods to correct for the selection bias and/or start of exposure and follow-up do not coincide, but the association of exposure is constant over time. **High risk of bias:** Selection into the study was related to exposure and outcome, or specific patient groups were selected for analysis, and this could not be corrected for in the analyses; or exposure and follow-up do not coincide and the effect of exposure (rate ratio) is not constant over time. No information: No information is reported about selection of participants into the study. |
| 4. Risk of bias due to post-exposure interventions | • Were there post-exposure interventions during follow-up that were influenced by prior dietary exposure and could affect the outcome? • If such interventions occurred, did the analysis appropriately account for their effects?  Notes: This domain assessed post-exposure interventions that were influenced by prior dietary exposure and could affect the outcome. Ordinary changes in habitual diet during follow-up and the absence of repeated dietary measurements were considered under Domain 2 (risk of bias arising from measurement of the exposure), rather than Domain 4. Based on the information reported, no relevant post-exposure intervention influenced by prior dietary exposure was identified in any of the 12 studies. Accordingly, Domain 4 was judged to be at low risk of bias in all studies. | **Low risk of bias:**  There were probably no relevant post-exposure interventions that were influenced by prior dietary exposure and could affect the outcome. **Some concerns (moderate risk of bias):**  Relevant post-exposure interventions influenced by prior dietary exposure occurred or probably occurred, and the analysis attempted to account for their effects, but some uncertainty remained. **High risk of bias:**  Relevant post-exposure interventions influenced by prior dietary exposure occurred or probably occurred, and the analysis did not appropriately account for their effects. No information: The available information was insufficient to determine whether a relevant post-exposure intervention occurred. |
| 5. Bias due to missing data | • Were there missing outcome data? • Were participants excluded due to missing data on exposure status? • Were participants excluded due to missing data on other variables needed for analysis?   Notes: Missing data on exposure variables and other variables are expected to be missing at random and not related to exposure or outcomes assessed during follow-up. | **Low risk of bias:**  Little loss-to-follow-up (<20%) and data on exposure and other variables were reasonably complete (<10% missing data) and were unlikely to introduce bias.  or the analysis addressed missing data and is likely to have removed any risk of bias. **Some concerns (moderate risk of bias):** There is a proportion of missing data in the original cohort or a high proportion of loss-to-follow-up and the analysis is unlikely to have removed the risk of bias arising from the missing data (e.g., using logistic regression). **High risk of bias:** High proportions (>50%) of missing data and the analysis is unlikely to have removed the risk of bias arising from the missing data  or missing data were addressed inappropriately in the analysis;  or the nature of the missing data means that the risk of bias cannot be removed through appropriate analysis. No information: No information is reported about missing data or the potential for data to be missing. |
| 6. Risk of bias due to measurement of the outcome | • Were the methods of outcome assessment comparable across exposure groups? • Could the outcome measure have been influenced by knowledge of the exposure status? • Was any systematic error in measurement of the outcome related to exposure status?   Notes: In observational studies, outcome assessors were not expected to be aware of participants’ exposure status. | **Low risk of bias:**  The methods of outcome assessment were comparable across exposure groups and the outcome measure was unlikely to be influenced by knowledge of the exposure status of study participants and any error in measuring the outcome is unrelated to exposure status (i.e. objective measures or self-reported outcomes that are (mostly, ≥90%) confirmed by a second source, e.g., medical records, record linkage and death certificates). **Some concerns (moderate risk of bias):** The methods of outcome assessment were comparable across exposure groups and any error in measuring the outcome may be minimally related to exposure status or outcomes were not reliably measured (i.e. confirmed records are available for <90% of all participants and the authors did not perform an additional analysis separating confirmed and probable cases). **High risk of bias:** The methods of outcome assessment were not comparable across exposure groups, or the outcome measure was subjective without confirmation by a second source, and error in measuring the outcome was related to exposure status. No information: No information is reported about the methods of outcome assessment. |
| 7. Risk of bias due to selection of the reported result | • Was the result reported in accordance with an available, pre-determined analysis plan?  • Is the reported effect estimate likely to be selected from multiple analyses of exposure-outcome relationship?  • Is the reported effect estimate likely to be selected from different subgroups?  Notes: In observational studies, it is unusual to publish an a priori analysis plan or protocol. Multiple outcome measurements for the definition of CVD, mortality, type 2 diabetes, etc. are not expected. | **Low risk of bias:**  There is a clear description of all analyses and the analyses are consistent, and all reported results correspond to all intended outcomes, analyses and sub-cohorts. **Some concerns (moderate risk of bias):** The results are reported according to an a priori analysis plan or protocol, and there is indication of selection of the reported analysis among multiple analyses; or there is indication of selection of the cohort or subgroups for analysis and reporting on the basis of the results (e.g. estimates not shown for all analyses). **High risk of bias:** There is a high risk of selective reporting from multiple exposure measurements, or outcome measurements, or multiple analyses of data or the cohort or subgroup is selected from a larger study for analysis and appears to be reported based on the results. No information: There is too little information to make a judgment. |
| Overall judgment | **Low risk of bias: The study is judged to be at low risk of bias for all domains. Some concerns (moderate risk of bias): The study is judged to be at low risk of bias or some concerns (moderate) for all domains. High risk of bias: The study is judged to be at high risk of bias in at least one domain, but no domains are at very high risk of bias. Very high risk of bias: The study is judged to be at very high risk of bias in at least one domain.** | |

Note. The domain-specific operational criteria were adapted from Supplemental Appendix 2 of Wallerer et al., based on the ROBINS-E framework described by Higgins et al., and modified for the present review of dietary-pattern studies. The source material was available under the Creative Commons Attribution 4.0 International License; changes were made.

**Supplementary Table 2. Risk-of-bias summary for studies included in the main meta-analyses and auxiliary food-group loading visualizations.**

| **Domain** | | **A priori dietary patterns** | | | **A posteriori dietary patterns** | | | | | | | | |
| --- | --- | --- | --- | --- | --- | --- | --- | --- | --- | --- | --- | --- | --- |
|  |  | Okada 2018 | Abe 2020 | Murai 2025 | Masaki 2003 | Kim 2004 | Kim 2005 | Shimazu 2007 | Pham 2010 | Maruyama 2013 | Kumagai 2014 | Nanri 2017 | Shin 2017 |
| Domain 1 | Risk of bias due to confounding | Some concerns | Some concerns | Some concerns | High risk | Some concerns | Some concerns | Some concerns | High risk | Some concerns | Some concerns | Some concerns | Some concerns |
| Domain 2 | Risk of bias arising from measurement of the exposure | Some concerns | Some concerns | Some concerns | High risk | Some concerns | Some concerns | Some concerns | Some concerns | Some concerns | Some concerns | Some concerns | Some concerns |
| Domain 3 | Risk of bias in selection of participants into the study (or into the analysis) | Low risk | Low risk | Low risk | Low risk | Low risk | Low risk | Low risk | Low risk | Low risk | Low risk | Low risk | Low risk |
| Domain 4 | Risk of bias due to post-exposure interventions | Low risk | Low risk | Low risk | Low risk | Low risk | Low risk | Low risk | Low risk | Low risk | Low risk | Low risk | Low risk |
| Domain 5 | Risk of bias due to missing data | Some concerns | High risk | Some concerns | Some concerns | Some concerns | Some concerns | Low risk | Some concerns | Some concerns | Some concerns | Some concerns | Some concerns |
| Domain 6 | Risk of bias arising from measurement of the outcome | Low risk | Low risk | Low risk | Low risk | Low risk | Low risk | Low risk | Low risk | Low risk | Low risk | Low risk | Low risk |
| Domain 7 | Risk of bias in selection of the reported result | Some concerns | Some concerns | Some concerns | Some concerns | Some concerns | Some concerns | Some concerns | Some concerns | Some concerns | Some concerns | Some concerns | Some concerns |
| **Overall** | | **Some concerns** | **High risk** | **Some concerns** | **High risk** | **Some concerns** | **Some concerns** | **Some concerns** | **High risk** | **Some concerns** | **Some concerns** | **Some concerns** | **Some concerns** |

**Supplementary Table 3. Reconstructed JDI-9 component loadings and similarity-based classification of a posteriori dietary patterns.**

| Study ID | First author, year (sex) | Dietary pattern name | JDI-9 components | | | | | | | | |  | Cosine similarity score | Cosine similarity category | Distance score | Distance score category | Classification |
| --- | --- | --- | --- | --- | --- | --- | --- | --- | --- | --- | --- | --- | --- | --- | --- | --- | --- |
|  |  |  | Rice | Miso/miso soup | Fish/shellfish | Vegetables | Seaweed | Pickles | Green tea | Meat | Coffee | Available JDI-9 components |  |  |  |  |  |
| 1 | Masaki et al., 2003  (Men) | Vegetable and fruit | 0.00 | 0.00 | 0.00 | 0.26 | 0.23 | 0.00 |  | 0.00 | 0.00 | 8 | 0.499 | Close | 0.939 | Far | Non-JDI-9-like DP |
|  |  | Western breakfast | -0.20 | -0.27 | -0.21 | -0.01 | -0.24 | 0.00 |  | 0.00 | 0.22 | 8 | -0.792 | Farthest | 1.143 | Farthest | Non-JDI-9-like DP |
|  |  | Meat | 0.00 | 0.00 | 0.00 | 0.00 | 0.00 | -0.21 |  | 0.39 | 0.00 | 8 | -0.480 | Farthest | 1.074 | Far | Non-JDI-9-like DP |
|  |  | Rice/snack | 0.30 | 0.27 | 0.00 | -0.19 | 0.00 | 0.00 |  | 0.00 | 0.00 | 8 | 0.305 | Far | 0.952 | Far | Non-JDI-9-like DP |
| 2 | Kim et al., 2004 (Men) | Healthy | 0.00 | 0.00 | 0.36 | 0.62 | 0.56 | 0.30 | 0.00 | 0.00 | 0.00 | 9 | 0.641 | Close | 0.796 | Close | JDI-9-like DP |
|  |  | Traditional | 0.42 | 0.43 | 0.43 | 0.00 | 0.00 | 0.57 | 0.00 | 0.00 | 0.00 | 9 | 0.661 | Closest | 0.794 | Close | JDI-9-like DP |
|  |  | Western | 0.00 | 0.00 | 0.00 | 0.00 | 0.00 | 0.00 | 0.00 | 0.46 | 0.21 | 9 | -0.442 | Farthest | 1.074 | Far | Non-JDI-9-like DP |
|  | Kim et al., 2004 (Women) | Healthy | 0.00 | 0.00 | 0.31 | 0.59 | 0.59 | 0.00 | 0.00 | 0.00 | 0.00 | 9 | 0.558 | Close | 0.834 | Close | JDI-9-like DP |
|  |  | Traditional | 0.51 | 0.50 | 0.48 | 0.00 | 0.00 | 0.65 | 0.00 | 0.00 | -0.31 | 9 | 0.728 | Closest | 0.728 | Closest | JDI-9-like DP |
|  |  | Western | 0.00 | 0.00 | 0.00 | 0.00 | 0.00 | 0.00 | 0.00 | 0.46 | 0.26 | 9 | -0.454 | Farthest | 1.080 | Farthest | Non-JDI-9-like DP |
| 3 | Kim et al., 2005 (Men) | Healthy | 0.00 | 0.00 | 0.36 | 0.62 | 0.56 | 0.30 | 0.00 | 0.00 | 0.00 | 9 | 0.641 | Close | 0.796 | Close | JDI-9-like DP |
|  |  | Traditional | 0.42 | 0.43 | 0.43 | 0.00 | 0.00 | 0.57 | 0.00 | 0.00 | 0.00 | 9 | 0.661 | Closest | 0.794 | Close | JDI-9-like DP |
|  |  | Western | 0.00 | 0.00 | 0.00 | 0.00 | 0.00 | 0.00 | 0.00 | 0.46 | 0.21 | 9 | -0.442 | Farthest | 1.074 | Far | Non-JDI-9-like DP |
|  | Kim et al., 2005 (Women) | Healthy | 0.00 | 0.00 | 0.31 | 0.59 | 0.59 | 0.00 | 0.00 | 0.00 | 0.00 | 9 | 0.558 | Close | 0.834 | Close | JDI-9-like DP |
|  |  | Traditional | 0.51 | 0.50 | 0.48 | 0.00 | 0.00 | 0.65 | 0.00 | 0.00 | -0.31 | 9 | 0.728 | Closest | 0.728 | Closest | JDI-9-like DP |
|  |  | Western | 0.00 | 0.00 | 0.00 | 0.00 | 0.00 | 0.00 | 0.00 | 0.46 | 0.26 | 9 | -0.454 | Farthest | 1.080 | Farthest | Non-JDI-9-like DP |
| 4 | Shimazu et al., 2007 | Japanese | 0.00 | 0.25 | 0.42 | 0.53 | 0.59 | 0.41 | 0.29 | 0.00 | 0.00 | 9 | 0.784 | Closest | 0.723 | Closest | JDI-9-like DP |
|  |  | DFA (high-dairy, high-fruit-and-vegetable, low-alcohol) | -0.59 | -0.39 | 0.00 | 0.11 | 0.26 | 0.00 | 0.00 | 0.00 | 0.00 | 9 | -0.266 | Far | 1.067 | Far | Non-JDI-9-like DP |
|  |  | Animal food | 0.00 | 0.00 | 0.11 | 0.00 | 0.00 | 0.00 | 0.00 | 0.50 | 0.29 | 9 | -0.387 | Far | 1.076 | Farthest | Non-JDI-9-like DP |
| 5 | Pham et al., 2010 (Men) | Vegetable | 0.00 | 0.31 | 0.22 | 0.52 | 0.62 | 0.35 | 0.00 | 0.00 | 0.00 | 9 | 0.702 | Closest | 0.775 | Closest | JDI-9-like DP |
|  |  | Animal food | 0.20 | 0.00 | 0.38 | 0.00 | 0.00 | 0.00 | 0.00 | 0.48 | -0.21 | 9 | 0.150 | Far | 0.966 | Far | Non-JDI-9-like DP |
|  |  | Dairy product | -0.21 | -0.25 | 0.00 | 0.00 | 0.00 | 0.00 | 0.00 | 0.09 | 0.41 | 9 | -0.602 | Farthest | 1.107 | Farthest | Non-JDI-9-like DP |
|  | Pham et al., 2010 (Women) | Vegetable | 0.00 | 0.33 | 0.23 | 0.50 | 0.62 | 0.25 | 0.00 | 0.00 | 0.00 | 9 | 0.694 | Closest | 0.786 | Closest | JDI-9-like DP |
|  |  | Animal food | 0.22 | 0.00 | 0.27 | 0.04 | 0.00 | 0.00 | 0.00 | 0.49 | 0.00 | 9 | 0.017 | Far | 0.997 | Far | Non-JDI-9-like DP |
|  |  | Dairy product | -0.24 | -0.29 | -0.07 | 0.00 | 0.00 | 0.00 | 0.00 | 0.09 | 0.26 | 9 | -0.671 | Farthest | 1.105 | Farthest | Non-JDI-9-like DP |
| 6 | Maruyama et al., 2013 (Men) | Vegetable | 0.05 | 0.35 | 0.26 | 0.52 | 0.63 | 0.35 | 0.12 | 0.05 | 0.03 | 9 | 0.734 | Closest | 0.756 | Closest | JDI-9-like DP |
|  |  | Animal food | 0.18 | 0.03 | 0.37 | 0.18 | 0.08 | 0.20 | 0.00 | 0.47 | -0.14 | 9 | 0.336 | Close | 0.922 | Close | JDI-9-like DP |
|  |  | Dairy product | -0.39 | -0.24 | -0.01 | 0.02 | 0.06 | 0.15 | 0.04 | 0.11 | 0.45 | 9 | -0.462 | Farthest | 1.104 | Farthest | Non-JDI-9-like DP |
|  | Maruyama et al., 2013 (Women) | Vegetable | -0.05 | 0.35 | 0.26 | 0.50 | 0.61 | 0.24 | 0.11 | 0.03 | -0.03 | 9 | 0.717 | Closest | 0.776 | Closest | JDI-9-like DP |
|  |  | Animal food | 0.22 | 0.07 | 0.34 | 0.13 | 0.06 | 0.17 | 0.05 | 0.46 | -0.01 | 9 | 0.297 | Far | 0.935 | Close | Non-JDI-9-like DP |
|  |  | Dairy product | -0.44 | -0.27 | -0.12 | 0.00 | 0.09 | 0.05 | 0.03 | 0.15 | 0.47 | 9 | -0.581 | Farthest | 1.142 | Farthest | Non-JDI-9-like DP |
| 7 | Kumagai et al., 2014 | Japanese | 0.00 | 0.00 | 0.42 | 0.53 | 0.60 | 0.41 | 0.29 | 0.00 | 0.00 | 9 | 0.725 | Closest | 0.750 | Closest | JDI-9-like DP |
|  |  | Animal food | 0.00 | 0.00 | 0.11 | 0.00 | 0.00 | 0.00 | 0.00 | 0.50 | 0.29 | 9 | -0.387 | Far | 1.076 | Farthest | Non-JDI-9-like DP |
|  |  | DFA (high-dairy, high-fruit-and-vegetable, low-alcohol) | -0.58 | -0.39 | 0.00 | 0.11 | 0.26 | 0.00 | 0.00 | 0.00 | 0.00 | 9 | -0.264 | Far | 1.066 | Far | Non-JDI-9-like DP |
| 8 | Nanri et al., 2017 | Traditional | 0.19 | 0.21 | 0.36 | 0.08 | 0.00 | 0.33 | 0.00 | 0.35 | 0.00 | 9 | 0.407 | Close | 0.909 | Close | JDI-9-like DP |
|  |  | Prudent | 0.00 | 0.13 | 0.35 | 0.60 | 0.68 | 0.51 | 0.37 | 0.09 | 0.00 | 9 | 0.728 | Closest | 0.717 | Closest | JDI-9-like DP |
|  |  | Westernized | 0.00 | 0.00 | 0.16 | 0.13 | 0.11 | -0.11 | 0.00 | 0.38 | 0.45 | 9 | -0.281 | Far | 1.060 | Far | Non-JDI-9-like DP |
| 9 | Shin et al., 2017 Men | Traditional | 0.22 | 0.17 | 0.33 | 0.04 | 0.00 | 0.48 | 0.18 | 0.14 | 0.00 | 9 | 0.619 | Close | 0.858 | Close | JDI-9-like DP |
|  | Shin et al., 2017 Men | Prudent | 0.00 | 0.17 | 0.30 | 0.65 | 0.71 | 0.45 | 0.35 | 0.14 | 0.00 | 9 | 0.706 | Closest | 0.723 | Closest | JDI-9-like DP |
|  | Shin et al., 2017 Men | Westernized | 0.00 | 0.00 | 0.25 | 0.09 | 0.00 | 0.00 | 0.00 | 0.44 | 0.39 | 9 | -0.252 | Far | 1.054 | Far | Non-JDI-9-like DP |
|  | Shin et al., 2017 Women | Traditional | 0.20 | 0.22 | 0.46 | 0.13 | 0.00 | 0.42 | 0.00 | 0.37 | 0.00 | 9 | 0.446 | Close | 0.882 | Close | JDI-9-like DP |
|  | Shin et al., 2017 Women | Prudent | 0.00 | 0.00 | 0.26 | 0.60 | 0.73 | 0.46 | 0.39 | 0.00 | 0.16 | 9 | 0.653 | Close | 0.747 | Closest | JDI-9-like DP |
|  | Shin et al., 2017 Women | Westernized | 0.00 | 0.00 | 0.14 | 0.03 | 0.00 | -0.15 | 0.00 | 0.38 | 0.47 | 9 | -0.434 | Far | 1.093 | Farthest | Non-JDI-9-like DP |

**Note:** Values under the JDI-9 components represent reconstructed food-group loadings mapped from the original a posteriori dietary pattern loadings. The JDI-9 reference profile consisted of 7 positive scoring items—rice, miso or miso soup, fish and shellfish, vegetables, seaweed, pickles, and green tea—and 2 reverse-scored components—meat and coffee. Cosine similarity was calculated by comparing each reconstructed JDI-9 component vector with the JDI-9 reference vector. Higher cosine similarity scores indicate greater similarity to the JDI-9 reference profile. The distance score represents the mean absolute distance from the JDI-9 reference profile; therefore, lower distance scores indicate greater similarity. For each metric, patterns were categorized into quartiles among the classifiable a posteriori dietary patterns. For cosine similarity, categories were labeled from highest to lowest similarity as Closest, Close, Far, and Farthest. For the distance score, categories were labeled from lowest to highest distance as Closest, Close, Far, and Farthest. Patterns classified as Closest or Close by both metrics were defined as JDI-9-like DPs. Patterns that did not meet both criteria were classified as Non-JDI-9-like DPs. Values of 0 indicate food groups that were assessed in the original study but whose loadings were either not reported or below the study-specific reporting threshold; these loadings were assigned a value of 0 for harmonization. Blank cells indicate unavailable components that could not be mapped from the original dietary assessment or reporting scheme. DP, dietary pattern; JDI-9, Japanese Diet Index-9.

**Supplementary Methods. Calculation of JDI-9 Similarity Metrics, Search Strategy, and Protocol Deviations**

To quantify the degree to which each a posteriori dietary pattern resembled the predefined JDI-9 reference profile, we calculated a cosine similarity score between the reconstructed JDI-9 component-loading vector of each dietary pattern and a JDI-9 reference vector. For component *j*, the reference value *rj* was set to +1 for positive scoring items and −1 for negative scoring items. The positive scoring items were rice, miso or miso soup, fish and shellfish, vegetables, seaweed, pickles, and green tea, whereas the negative scoring items were meat and coffee.

Let $x_{pj}$ denote the reconstructed loading of component $j$ in dietary pattern $p$, and let $A_{p}$ denote the set of JDI-9 components available for dietary pattern $p$. The cosine similarity score was calculated as follows:

$$\text{Cosine similarity score}_{p}=\frac{\sum_{j\in A_{p}} x_{pj}r_{j}}{\sqrt{\sum_{j\in A_{p}} x_{pj}^{2}}\sqrt{\sum_{j\in A_{p}} r_{j}^{2}}}$$

Higher cosine similarity scores indicate greater similarity to the JDI-9 reference profile.

As a complementary metric, we calculated a distance score from the same reference vector:

$$\text{Distance score}_{p}=\frac{1}{\mid A_{p}\mid}\sum_{j\in A_{p}} \mid x_{pj}-r_{j}\mid$$

Lower distance scores indicate greater similarity to the JDI-9 reference profile. For both metrics, only available JDI-9 components were included in the calculation. Loadings assigned a value of 0 because the food group was assessed but its loading was not reported in the original loading table were included in the calculation, whereas unavailable components that could not be mapped from the original dietary assessment or reporting scheme were excluded. For each metric, dietary patterns were categorized into quartiles among the included a posteriori dietary patterns. For the cosine similarity score, quartiles were labeled from highest to lowest similarity as Closest, Close, Far, and Farthest. For the distance score, quartiles were labeled from lowest to highest distance using the same terms. In the absence of an externally validated cutoff, the Closest and Close categories were used as an exploratory distribution-based threshold. The primary classification used a conservative conjunctive rule: patterns classified as Closest or Close by both metrics were defined as JDI-9-like dietary patterns. As a sensitivity check, the Closest or Close criterion was also applied separately to cosine similarity and to the distance score. This classification was used as a relative food-structural annotation for visualization and interpretation, not as an externally validated definition of a healthful dietary pattern.

**Supplementary text. Search strategy**

**1. PubMed/MEDLINE** (Initial search: 24 February 2026; final search: 14 March 2026)

("Japanese Diet Index"[tiab] OR "Modified Japanese Diet Index"[tiab] OR jdi[tiab] or jdi8[tiab] OR jdi9[tiab] or jdi12[tiab] OR mjdi*[tiab] OR wjdi*[tiab] OR "Japanese diet"[tiab] OR "Japanese dietary pattern"[tiab] OR "Japanese dietary patterns"[tiab] OR "traditional Japanese diet"[tiab] OR washoku*[tiab] OR ("Japan"[Mesh] AND "Diet"[Mesh]))

*AND*

("mortality"[tiab] OR "all-cause mortality"[tiab] OR "cause-specific mortality"[tiab] OR "cardiovascular disease*"[tiab] OR "cardiovascular mortality"[tiab] OR "cancer mortality"[tiab] OR "cerebrovascular disease*"[tiab] OR cvd[tiab] OR stroke[tiab] OR "ischemic stroke"[tiab] OR "hemorrhagic stroke"[tiab] OR "coronary heart disease"[tiab] OR "ischemic heart disease"[tiab] OR "myocardial infarction"[tiab] OR cancer[tiab] OR dementia[tiab] OR "Alzheimer's disease"[tiab] OR "vascular dementia"[tiab] OR "cognitive decline"[tiab] OR "cognitive impairment"[tiab] OR "cognitive function"[tiab] OR "mild cognitive impairment"[tiab] OR MCI[tiab] OR "Mini-Mental State Examination"[tiab] OR MMSE[tiab] OR hypertension[tiab] OR "high blood pressure"[tiab] OR SBP[tiab] OR DBP[tiab] OR "systolic blood pressure"[tiab] OR "diastolic blood pressure"[tiab] OR frailty[tiab] OR fall*[tiab] OR fracture*[tiab] OR disability[tiab] OR "functional decline"[tiab] OR "activities of daily living"[tiab] OR ADL[tiab] OR sarcopenia[tiab] OR "muscle mass"[tiab] OR "long-term care"[tiab] OR "care need"[tiab] OR ("Cardiovascular Diseases"[Mesh] OR "Neoplasms"[Mesh] OR "Cerebrovascular Disorders"[Mesh] OR "Dementia"[Mesh] OR "Cognition Disorders"[Mesh] OR "Frailty"[Mesh] OR "Hypertension"[Mesh]) )

*AND*

(cohort*[tiab] OR prospective[tiab] OR longitudinal[tiab] OR "follow-up"[tiab] OR "case-control"[tiab] OR "cross-sectional"[tiab] OR trial[tiab] OR randomized[tiab] OR observational[tiab] OR epidemiologic[tiab] OR ("Cohort Studies"[Mesh] OR "Case-Control Studies"[Mesh] OR "Cross-Sectional Studies"[Mesh] OR "Randomized Controlled Trials as Topic"[Mesh]))

*NOT*

(animals[mh] NOT humans[mh])

*NOT*

(review[pt] OR meta-analysis[pt] OR case reports[pt] OR editorial[pt] OR comment[pt])

**2.Web of Science Core Collection** (Initial search: 24 February 2026; final search: 14 March 2026)

Topic

("Japanese Diet Index" OR "Modified Japanese Diet Index" OR jdi OR jdi8 OR jdi9 OR jdi12 OR mjdi* OR wjdi* OR "Japanese diet" OR "Japanese dietary pattern" OR "Japanese dietary patterns" OR "traditional Japanese diet" OR washoku* OR ("Japan" AND "Diet"))

*AND*

Topic

("mortality" OR "all-cause mortality" OR "cause-specific mortality" OR "cardiovascular disease*" OR "cardiovascular mortality" OR "cancer mortality" OR "cerebrovascular disease*" OR cvd OR stroke OR "ischemic stroke" OR "hemorrhagic stroke" OR "coronary heart disease" OR "ischemic heart disease" OR "myocardial infarction" OR cancer OR dementia OR "Alzheimer's disease" OR "vascular dementia" OR "cognitive decline" OR "cognitive impairment" OR "cognitive function" OR "mild cognitive impairment" OR MCI OR "Mini-Mental State Examination" OR MMSE OR hypertension OR "high blood pressure" OR SBP OR DBP OR "systolic blood pressure" OR "diastolic blood pressure" OR frailty OR fall* OR fracture* OR disability OR "functional decline" OR "activities of daily living" OR ADL OR sarcopenia OR "muscle mass" OR "long-term care" OR "care need" OR ("Cardiovascular Diseases" OR "Neoplasms" OR "Cerebrovascular Disorders" OR "Dementia" OR "Cognition Disorders" OR "Frailty" OR "Hypertension"))

*AND*

Topic

(cohort* OR prospective OR longitudinal OR "follow-up" OR "case-control" OR "cross-sectional" OR trial OR randomized OR observational OR epidemiologic OR ("Cohort Studies" OR "Case-Control Studies" OR "Cross-Sectional Studies" OR "Randomized Controlled Trials as Topic"))

Document Types filter: Article only.

**Supplementary Table 4. PECOTS-based review scope and requirements for the analytic syntheses.**

| **PECOTS domain** | **Registered scope, review-specific selection, and qualitative reporting** | **Additional eligibility for the present analytic synthesis** |
| --- | --- | --- |
| **Population** | The registered population comprised general community-dwelling residents of Japan without target diseases, with no age restrictions. Studies of Japanese-origin populations residing outside Japan and studies in which extraordinary circumstances could prevent dietary assessment from reflecting usual habitual intake were excluded. For qualitative reporting, the memory-clinic-based study was also retained because it evaluated Japanese-style dietary indices across cognitive-status groups. Its clinical recruitment setting was explicitly identified and was not assumed to represent a general population sample. | Same as for the broad review, except that the memory-clinic-based report was retained for qualitative reporting only. |
| **Exposure** | Eligible exposures were predefined (*a priori*) Japanese-style DP scores or food-based component-score DPs derived using principal component analysis (PCA) or factor analysis (FA). Food-based reduced rank regression (RRR) patterns were retained for qualitative synthesis. Data-derived exposures defined by cluster membership after nonlinear embedding followed by clustering were outside the review-specific exposure scope. The DP itself had to be evaluated in relation to the health outcome. Individual-food or nutrient exposures, nutrient-derived patterns, and broader lifestyle indices in which a diet score was only one component were excluded. | **A priori stream:** Japanese-style DP scores providing comparable adjusted estimates for the same mortality outcomes and permitting highest-versus-lowest study-specific score contrasts. **A posteriori stream:** PCA- or FA-derived DPs with sufficient reported food-group loading information for reconstruction and visualization. |
| **Comparator** | A lower study-defined category or score of the same DP, or a continuous contrast in the same DP score or factor score. | **A priori stream:** highest versus lowest study-specific DP score categories.  **A posteriori stream:** highest-versus-lowest study-specific dietary-pattern categories with an extractable adjusted effect estimate and 95% CI. |
| **Outcomes** | Preregistered main outcomes: all-cause mortality; cardiovascular disease; stroke; cancer; long-term care need; decline in activities of daily living or instrumental activities of daily living (ADL/IADL); cognitive function; falls or fractures; sarcopenia; and frailty. Reports evaluating related cardiometabolic measures and conditions, including blood pressure, hypertension, serum lipids, glycemic measures, adiposity, and inflammatory markers, were retained as additional descriptive evidence in the qualitative synthesis. These outcomes were not treated as preregistered outcomes and did not contribute to the present analytic synthesis. Premalignant lesions and composite measures such as disability-free survival time or disability-adjusted life years remained outside the selected outcome scope unless an eligible outcome was reported separately. All registered outcomes were treated as main outcomes; no secondary-outcome hierarchy was prespecified. | **A priori meta-analysis:** all-cause mortality, CVD mortality, and total cancer mortality.  **A posteriori visualization:** all-cause mortality, CVD mortality, gastric cancer incidence or mortality, and colorectal cancer incidence. |
| **Timing** | Cross-sectional assessment or longitudinal follow-up; no minimum follow-up duration was prespecified. | Longitudinal follow-up in which dietary exposure was assessed before outcome occurrence. |
| **Study design** | Peer-reviewed full-text original nonrandomized cross-sectional or cohort studies reporting individual-level data. Reviews, conference abstracts without a full peer-reviewed article, ecological or aggregate-level international comparative studies, and randomized or other intervention studies were excluded. | Longitudinal observational cohort studies. Cross-sectional studies were not included in the present analytic synthesis. |

**Note:** The registered scope and additional qualitative reporting are distinguished from the requirements of the analytic syntheses. The review-specific exposure-method criterion was applied at full-text assessment. The review-specific criterion focused the synthesis on score-defined dietary exposures, allowing findings to be interpreted in terms of graded differences in dietary-pattern scores and the food groups that define them. Nonlinear embedding followed by clustering instead defines exposure through membership of dietary-profile groups, which was treated as a different exposure construct outside this score-based scope. During data extraction and before the final synthesis, the analytic requirements were specified in response to differences in exposure definitions, outcome types, study designs, and the availability of adjusted estimates and food-group loadings. Longitudinal follow-up preserved temporal ordering; comparable scores and contrasts were required for pooling, and sufficient PCA/FA-derived loadings were required for graphical comparison. The selected analytic outcomes are listed in the right-hand column. Review-specific full-text selection decisions are documented in Supplementary Table 5. Findings from the other 25 retained reports, including cross-sectional and food-based RRR evidence, are summarized in Supplementary Table 6, with risk-of-bias judgments in Supplementary Table 7.

**Supplementary Table 5. Reports excluded after full-text assessment and primary reasons for exclusion.**

| **No.** | **Author, years** | **Study title** | **Primary PECOTS domain** | **Reason for exclusion** |
| --- | --- | --- | --- | --- |
| 1 | Marr and Morris, 1981 | Dietary intake and the risk of coronary heart disease in Japanese men living in Hawaii | Population | The study population consisted of Japanese men residing outside Japan. |
| 2 | Mizoue et al., 2005 | Dietary patterns and colorectal adenomas in Japanese men: the Self-Defense Forces Health Study | Outcomes | The outcome was colorectal adenoma rather than an eligible cancer incidence or mortality outcome. |
| 3 | Kiyohara, 2014 | Epidemiology of dementia: the Hisayama Study | Study design | The report was a review article and did not present an eligible original observational analysis. |
| 4 | Kanauchi and Kanauchi, 2017 | Development and nutritional validity of a novel Japanese diet score and its relation with health status | Exposure | The report primarily developed and nutritionally validated a Japanese diet score. Adjusted associations with hypertension and diabetes were reported for the composite Healthy 5 score, which included Japanese-diet adherence as one component, rather than for the Japanese diet score alone. Unadjusted comparisons of the Japanese diet score across health-status groups were also reported. |
| 5 | Iwasaki et al., 2019 | Associations of Nutrient Patterns with the Prevalence of Metabolic Syndrome: Results from the Baseline Data of the Japan Multi-Institutional Collaborative Cohort Study | Exposure | The empirically derived exposure represented nutrient-intake patterns rather than food-based dietary patterns. |
| 6 | Zhang et al., 2019 | The Japanese Dietary Pattern Is Associated with Longer Disability-Free Survival Time in the General Elderly Population in the Ohsaki Cohort 2006 Study | Outcomes | The outcome was disability-free survival time, a composite measure of survival and disability-free time, rather than one of the prespecified broad-review outcomes. |
| 7 | Ma et al., 2020 | Associations between Dietary Patterns and Cardiometabolic Risks in Japan: A Cross-Sectional Study from the Fukushima Health Management Survey, 2011–2015 | Population | The study assessed residents of evacuation areas after the Great East Japan Earthquake, with dietary assessments conducted during 2011–2013. The reported exclusion concerned this disaster-affected population context, which the review treated as outside its intended scope of usual living conditions. |
| 8 | Imai et al., 2024 | The Association Between the Japanese Diet Score and Years of Survival with Disability and Disability-Adjusted Life Years: A Longitudinal Study Using International Open Data | Study design | The report was a conference abstract, and no publicly available peer-reviewed full-text original article containing sufficient information for eligibility assessment was identified. |
| 9 | Li et al., 2024 | Dietary patterns associated with the incidence of hypertension among adult Japanese males: application of machine learning to a cohort study | Exposure | Dietary patterns were defined by UMAP followed by K-means clustering and described using trend scores. This cluster-membership exposure derived from a nonlinear embedding did not meet the review-specific data-derived exposure criterion in Supplementary Table 4. |

**Note.** This table documents the nine full-text exclusions and the review-specific judgments underlying them. These judgments are distinguished from the registered scope summarized in Supplementary Table 4. Of 46 reports assessed, 37 were retained: 12 contributed to the analytic syntheses and 25 to qualitative synthesis only. The latter reports are described in Supplementary Tables 6 and 7. The original Japanese title of Imai et al. (2024) was translated by the review authors.

**Supplementary Table 6. Characteristics and findings of the 25 reports included in qualitative synthesis only.**

| **First author, year (sex)** | **Study design** | **Population** | **Dietary-pattern approach / derivation** | **Outcome** | **Main findings** | **Reason for qualitative synthesis only** |
| --- | --- | --- | --- | --- | --- | --- |
| **A. Reports evaluating a priori dietary patterns, including reports evaluating both approaches** | | | | | | |
| Nakamura, 2009 (Both) [S1] | 19-y prospective cohort study. | n = 9,086 adults aged ≥30 y in Japan; NIPPON DATA80 (sample size stated in the Abstract and Methods). | A priori: 7-component healthy Reduced-Salt Japanese Diet Score, derived from an FFQ; food-intake components, low-salt soy-sauce use, infrequent noodle-soup consumption, and occasional drinking. | All-cause and cause-specific mortality. | In multivariable Model 2, scores 4–7 versus 0–2 were associated with HRs of 0.78 (95% CI 0.70–0.88) for all-cause mortality, 0.80 (0.66–0.97) for CVD mortality, and 0.85 (0.69–1.05) for cancer mortality. | Score construction combined food intake with salt-reduction practices and drinking behavior, limiting comparability with the pooled indices. |
| Tomata, 2014 (Both) [S2] | 5-y prospective cohort study. | Adults aged ≥65 y; Ohsaki Cohort 2006, Miyagi, Japan. PCA analysis: n = 14,260; Japanese Diet Index analysis: n = 10,148. | A priori and a posteriori: 9-component Japanese Diet Index (JDI; 0–9); PCA of 39 FFQ food/beverage items, yielding Japanese, Animal food, and High dairy patterns. | Incident functional disability, defined by Long-term Care Insurance (LTCI) certification. | For the JDI, the highest versus lowest quartile HR for incident functional disability was 0.79 (95% CI 0.68–0.92; P for trend = 0.002). In PCA Model 3, corresponding HRs were 0.77 (0.68–0.88; P for trend <0.001) for Japanese, 1.16 (1.02–1.31; P for trend = 0.053) for Animal food, and 1.11 (0.99–1.26; P for trend = 0.158) for High dairy patterns. | Functional disability was outside the outcomes selected for meta-analysis and visualization. |
| Tomata, 2016 (Both) [S3] | 5.7-y prospective cohort study. | Adults aged ≥65 y; Ohsaki Cohort 2006, Miyagi, Japan; n = 14,402 in the main PCA analysis. | A priori and a posteriori: 9-component JDI; PCA of 39 FFQ food/beverage items, yielding Japanese, Animal food, and High-dairy patterns. | Incident dementia, identified through the LTCI system. | In Model 3, the highest versus lowest JDI quartile HR for incident dementia was 0.79 (95% CI 0.66–0.95; P for trend = 0.015), as reported in the Results. The corresponding PCA Japanese-pattern HR was 0.80 (0.66–0.97; P for trend = 0.016). Animal food and High-dairy patterns were not significantly associated with dementia. | Dementia was outside the outcomes selected for meta-analysis and visualization. |
| Matsuyama, 2020 (Both) [S4] | Prospective cohort study with repeated dietary assessment; 10-y outcome follow-up. | n = 2,923 adults aged ≥65 y in 2006; linked Ohsaki surveys, Miyagi, Japan; dietary assessments in 1994 and 2006. | A priori: 9-component JDI from a 39-item FFQ; exposure was the 12-y change in score, classified into five categories. | Incident functional disability, defined by LTCI certification. | In Model 3, an increase of ≥2 JDI points versus a decrease of ≥2 points was associated with a lower hazard of incident functional disability (HR 0.77, 95% CI 0.61–0.98; P for trend = 0.045). | Score-change contrast rather than score level; functional disability was outside the selected analytic outcomes. |
| Suthuvoravut, 2020 (Both) [S5] | Cross-sectional study. | n = 1,241 community-dwelling adults aged ≥65 y who were not eligible for long-term care; Kashiwa Study, Chiba, Japan. | A priori and a posteriori: 7-food-group Japanese diet score; PCA of 47 BDHQ items, yielding Japanese side dishes, Japanese main dishes, and Noodles patterns. | Prevalent sarcopenia defined by AWGS criteria. | In Model 2, low versus high Japanese diet score was associated with sarcopenia in men (AOR 5.10, 95% CI 1.27–20.3); the corresponding estimate in women was 2.90 (0.95–8.88). Lowest versus highest Japanese-side-dish tertile in men: AOR 3.67 (1.20–11.2). Japanese-main-dish estimate in women: 2.71 (0.99–7.46). | Cross-sectional design; meta-analysis and visualization required longitudinal data. |
| Matsuyama, 2021 (Both) [S6] | Prospective cohort study. | n = 92,969 adults aged 45–74 y; JPHC Study, Japan; median follow-up 18.9 y. | A priori: 8-item Japanese Diet Index (JDI8; 0–8), assessed using a 147-item FFQ. | All-cause and cause-specific mortality. | In Model 3, JDI8 scores of 6–8 versus 0–2 were associated with HRs of 0.86 (95% CI 0.81–0.90) for all-cause mortality, 0.89 (0.80–0.99) for CVD mortality, and 0.94 (0.86–1.03) for cancer mortality. | Overlapping JPHC mortality data; the more recent report by Murai et al. (2025) contributed to pooling. |
| Lu, 2021 (Both) [S7] | 5.7-y prospective cohort study with repeated dietary assessment. | n = 3,146 adults aged ≥65 y in 2006; linked Ohsaki surveys, Miyagi, Japan; dietary assessments in 1994 and 2006. | A priori: JDI8 from a 39-item FFQ; exposure was 12-y score change, classified as ≤−2, −1, 0, +1, or ≥+2 points. | Incident disabling dementia, identified through the LTCI system. | In Model 3, versus no score change, HRs were 1.72 (95% CI 1.13–2.62) for a change of ≤−2 points, 1.10 (0.73–1.66) for −1 point, 0.82 (0.54–1.25) for +1 point, and 0.62 (0.38–1.02) for ≥+2 points; P for trend <0.0001 (Table 2). | Score-change contrast rather than score level; dementia was outside the selected analytic outcomes. |
| Saji, 2022 (Both) [S8] | Cross-sectional subanalysis of a hospital-based prospective cohort. | n = 85 memory-clinic patients; Gimlet Study, National Center for Geriatrics and Gerontology, Japan; mean age 74.6 y; 23 had dementia. | A priori: JDI9, JDI12, and revised rJDI12. JDI12 adds soy foods, fruit, and mushrooms to JDI9; rJDI12 additionally scores coffee positively. | Dementia prevalence, cognitive function, gut microbiota, and fecal microbial metabolites. | In multivariable Model 4, high versus low score ORs for dementia were 0.36 (95% CI 0.12–1.03) for JDI9, 0.10 (0.01–0.45) for JDI12, and 0.34 (0.07–1.32) for rJDI12. Most comparisons of microbial metabolites by score group were not statistically significant. | Cross-sectional design; meta-analysis and visualization required longitudinal data. |
| Zhang, 2023 (Both) [S9] | Prospective cohort study; follow-up up to 20 y. | n = 1,504 dementia-free adults aged 65–82 y; NILS-LSA, Aichi, Japan; median follow-up 11.4 y; dementia within the first 5 y was excluded. | A priori: weighted 9-component Japanese Diet Index (wJDI9; −1 to 12), assessed using 3-d dietary records. | Incident dementia and differences in age at dementia onset. | Highest versus lowest wJDI9 tertile: adjusted HR for incident dementia 0.58 (95% CI 0.40–0.86). The estimated 11th percentile of age at dementia onset was 36.7 months higher (95% CI 9.9–63.4). | Weighted score construction differed from the pooled indices; dementia was outside the selected analytic outcomes. |
| Miyake, 2025 (Both) [S10] | Cross-sectional study. | n = 12,213 employees at five Japanese companies; J-ECOH Study; mean age 42.2 y; 11.9% women. | A priori: 9-item Traditional Japanese Diet Score and a Modified Japanese Diet Score, from a 28-item FFQ; modifications included whole-grain rice, reversed scoring for salty foods, and added fruit, raw vegetables, and dairy. | Hypertension prevalence: SBP ≥140 mmHg, DBP ≥90 mmHg, or antihypertensive medication. | In multivariable Model 2, highest versus lowest quartile PRs were 0.96 (95% CI 0.90–1.02; P for trend = 0.29) for the Traditional score and 0.94 (0.87–1.01; P for trend = 0.10) for the Modified score. | Cross-sectional design; meta-analysis and visualization required longitudinal data. |
| **B. Reports evaluating only data-derived dietary patterns** | | | | | | |
| Kumagai, 1999 (Both) [S11] | 7-y longitudinal cohort study. | n = 630 community-dwelling adults aged ≥65 y; Nangai Village, Akita, Japan (total stated in the Methods and Table 1). | A posteriori: FA of 15 FFQ food groups; four factors characterized by plant foods; meat, fat and oil; bread and rice (negative); and salty pickled vegetables, fish and shellfish. | All-cause mortality. | The continuous plant-food factor score was inversely associated with 7-year all-cause mortality (adjusted RR, as reported, 0.77; 95% CI 0.63–0.95). The other three factor scores were not significantly associated with mortality. Estimates were obtained from a multiple logistic regression model. | Continuous factor-score estimates only; no highest-versus-lowest category estimate for visualization. |
| Sadakane, 2008 (Both) [S12] | Cross-sectional analysis of cohort baseline data. | Adults aged 40–69 y in the Jichi Medical School Cohort Study, Japan; n = 6,886 for blood pressure and n = 7,641 for serum lipids. | A posteriori: factor analysis using PCA with varimax rotation of 30 FFQ items; Vegetable, Meat, and Western patterns. | Blood pressure and serum total, HDL, and LDL cholesterol. | After adjustment, the Vegetable pattern was associated with lower SBP, DBP, and pulse pressure and higher HDL cholesterol in women. The Meat pattern was associated with higher total and HDL cholesterol in both sexes and higher LDL cholesterol in men. The Western pattern was associated with higher total and LDL cholesterol in both sexes. | Cross-sectional design and cardiometabolic intermediate outcomes outside the selected analytic scope. |
| Monma, 2010 (Both) [S13] | 4-y population-based prospective cohort study. | n = 877 community-dwelling adults aged ≥70 y; Tsurugaya district, Sendai, Japan; 28 incident fall-related fractures during follow-up. | A posteriori: factor analysis of 27 food groups from an FFQ; Vegetable, Meat, and Traditional Japanese patterns. | Incident fall-related fracture. | In Model 4, highest versus lowest tertile HRs were 2.62 (95% CI 0.93–7.41; P for trend = 0.044) for Vegetable, 0.36 (0.12–1.06; P for trend = 0.057) for Meat, and 0.76 (0.26–2.19; P for trend = 0.579) for Traditional Japanese. For Meat, the middle versus lowest tertile HR was 0.36 (0.14–0.96). | Fall-related fractures were outside the outcomes selected for visualization. |
| Ozawa, 2013 (Both) [S14] | Prospective cohort study; median follow-up 15 y. | n = 1,006 community-dwelling adults aged 60–79 y without dementia; Hisayama Study, Japan; 271 incident dementia cases. | Data-derived: reduced rank regression (RRR) of 19 food groups from a 70-item FFQ, with seven nutrients as response variables; seven patterns were derived, with the first pattern emphasized. | Incident all-cause dementia, Alzheimer disease, and vascular dementia. | For the first pattern, highest versus lowest quartile adjusted HRs were 0.66 (95% CI 0.46–0.95) for all-cause dementia, 0.65 (0.40–1.06) for Alzheimer disease, and 0.45 (0.22–0.91) for vascular dementia. The pattern emphasized soy foods, vegetables, algae, and dairy, with lower rice intake. | RRR-derived pattern rather than PCA/FA; dementia was outside the selected visualization outcomes. |
| Sugawara, 2015 (Both) [S15] | Cross-sectional study. | n = 388 community-dwelling adults aged ≥60 y; Iwaki Health Promotion Project 2011, Japan. | A posteriori: PCA of 52 food/beverage items from the BDHQ; Healthy, Noodle, and Alcohol and accompaniment patterns. | Poor cognitive function, defined in the Methods as MMSE ≤24. | Healthy and Noodle patterns were not significantly associated with poor cognitive function. For Alcohol and accompaniment, the fully adjusted middle versus lowest tertile OR was 2.85 (95% CI 1.04–7.80); the highest versus lowest tertile was not significantly associated. The Results did not support a dose-dependent association. | Cross-sectional design; meta-analysis and visualization required longitudinal data. |
| Niu, 2016 (Both) [S16] | 3-y longitudinal observational study. | n = 980 apparently healthy adults undergoing health examinations at the Sendai Oroshisho Center, Japan; 735 men and 245 women. | A posteriori: PCA with varimax rotation of 39 food groups; Japanese, Sweets-fruits-cooked wheaten food, and Izakaya (Japanese Pub) patterns. | Longitudinal changes in BMI, blood pressure, fasting blood glucose, lipids, and hs-CRP. | Greater Japanese-pattern adherence was associated with smaller increases in DBP in men (P for trend = 0.03) and in SBP and DBP in women (P for trend <0.01 and 0.04). The Izakaya pattern was associated with greater triglyceride change in men (P for trend = 0.02). The Sweets-fruits-cooked wheaten food pattern was not associated with these changes. | Changes in cardiometabolic risk factors were outside the mortality and cancer outcomes selected for the analytic syntheses. |
| Shin, 2016 (Women) [S17] | Prospective cohort study. | n = 49,552 Japanese women; JPHC Study; mean follow-up 14.6 y; 718 incident breast cancers. | A posteriori: PCA with varimax rotation of 48 food groups from the FFQ; Prudent, Westernized, and Traditional Japanese patterns. | Incident breast cancer. | Westernized pattern Q5 vs Q1: HR 1.32 (95% CI 1.03–1.70; P-trend=0.04). Prudent and Traditional Japanese patterns were not associated with breast cancer risk. | Breast cancer incidence was outside the outcomes selected for visualization. |
| Okada, 2016 (Men) [S18] | Prospective cohort study; median follow-up 18.9 y. | n = 26,562 men aged 40–79 y; JACC Study, Japan; follow-up during 1988–2009; 132 esophageal-cancer deaths. | A posteriori: factor analysis of 39 FFQ food items with varimax rotation; Vegetable, Animal food, and Dairy product patterns. | Esophageal cancer mortality. | Multivariable highest versus lowest quartile HRs were 0.81 (95% CI 0.48–1.36) for Vegetable, 1.15 (0.65–2.04) for Animal food, and 0.67 (0.38–1.18; P for trend = 0.042) for Dairy product. In subgroup analyses, the Dairy-product HR in smokers was 0.57 (0.30–1.09); the Animal-food HR in nonsmokers was 6.01 (1.17–30.88). | Esophageal cancer mortality was outside the outcomes selected for visualization. |
| Okubo, 2017 (Both) [S19] | Cross-sectional study. | n = 635 community-dwelling adults aged 69–71 y; baseline SONIC Study, Japan. | A posteriori: factor analysis of 33 food groups; Plant foods and fish, Rice and miso soup, and Animal food patterns. | Cognitive function assessed by MoCA-J. | Plant-foods-and-fish pattern was positively associated with MoCA-J score after adjustment (β per one-quartile increase 0.41, 95% CI 0.17–0.65; P-trend<0.001). Other patterns were not associated. | Cross-sectional design; meta-analysis and visualization required longitudinal data. |
| Kojima, 2017 (Women) [S20] | Prospective cohort study. | n = 23,172 Japanese women; JACC Study; median follow-up 16.9 y; 119 incident breast cancers. | A posteriori: factor analysis; Vegetable, Animal-food, and Dairy-product patterns. | Incident breast cancer. | Vegetable and Dairy-product patterns were not significantly associated with breast cancer. For Animal food, the multivariable highest versus lowest tertile HR was 0.42 (95% CI 0.18–0.93; P for trend = 0.04) in premenopausal women and 0.98 (0.48–1.99) in postmenopausal women. | Breast cancer incidence was outside the outcomes selected for visualization. |
| Htun, 2017 (Both) [S21] | Cross-sectional analysis. | n = 11,365 Japanese adults aged 20–84 y; 2012 National Health and Nutrition Survey; 4,686 men and 6,679 women. | A posteriori: PCA with varimax rotation of 34 food groups reorganized from the survey’s 98 groups; one-day semi-weighed dietary records; Traditional Japanese, Westernized, and Meat and fat patterns. | Adiposity, blood pressure, HbA1c and lipids; prevalence of hypertension, diabetes, high total cholesterol, and elevated LDL cholesterol. | The Traditional Japanese pattern was associated with lower hypertension prevalence in men (adjusted Q4 vs Q1 OR 0.67, 95% CI 0.53–0.84), but higher waist circumference/BMI in men and DBP in women. Westernized-pattern ORs in women were 1.80 (1.41–2.29) for hypercholesterolemia and 1.75 (1.30–2.35) for elevated LDL cholesterol. Meat and fat was positively associated with hypertension, diabetes, and hypercholesterolemia in men. | Cross-sectional design; meta-analysis and visualization required longitudinal data. |
| Shin, 2018 (Men) [S22] | Prospective cohort study; mean follow-up 13.8 y. | n = 43,469 Japanese men; JPHC Study; 1,156 incident prostate cancers. | A posteriori: exploratory factor analysis; Prudent, Westernized, and Traditional patterns. | Total, localized, and advanced prostate cancer incidence. | Westernized-pattern highest versus lowest quintile HRs were 1.22 (95% CI 1.00–1.49; P for trend = 0.021) for total prostate cancer, 1.24 (0.97–1.57) for localized disease, and 1.23 (0.82–1.84) for advanced disease. Prudent and Traditional patterns were not significantly associated with total prostate cancer in the overall analysis. | Prostate cancer incidence was outside the outcomes selected for visualization. |
| Zhao, 2019 (Both) [S23] | Prospective cohort study. | n = 2,949 community-dwelling adults aged 64–65 y; NISSIN Project, Japan; 31,233 person-years; 253 deaths. | A posteriori: exploratory factor analysis with varimax rotation of 20 food groups aggregated from 90 FFQ food items, yielding Meat-fat, Healthy, and Dairy-bread patterns. | All-cause mortality. | In Table 3, Model 3, highest versus lowest tertile HRs were 1.25 (95% CI 0.84–1.88) for Meat-fat, 0.74 (0.53–1.02) for Healthy, and 1.34 (0.98–1.83) for Dairy-bread; corresponding linear trends were not statistically significant. An inverse association was reported for the middle Healthy-pattern tertile. | The reported food-group scheme provided insufficient JDI-9 component detail for reconstruction and comparison. |
| Huang, 2021 (Both) [S24] | 3-y prospective cohort study with annual repeated outcome assessment. | n = 666 community-dwelling adults aged ≥60 y; Nagoya Longitudinal Study for Healthy Elderly, Japan. | A posteriori: PCA of 20 food groups; Mediterranean-style, Sugar-and-fat, Salt-and-pickles, Noodle-and-alcohol, and Protein-rich patterns. | Frailty, assessed using repeated measurements of a 54-item Frailty Index over 3 y. | In Model 2 of the longitudinal analysis, highest versus lowest quartile mean differences in the Frailty Index were +0.017 (95% CI 0.006–0.029) for Sugar and fat, +0.014 (0.003–0.025) for Salt and pickles, and −0.013 (−0.025 to −0.002) for Protein-rich patterns. Mediterranean-style and Noodle-and-alcohol patterns were not significantly associated. | Frailty trajectories were outside the outcomes selected for visualization. |
| Chen, 2026 (Both) [S25] | Cross-sectional multicenter study. | n = 8,938 adults aged ≥65 y; JPSC-AD, eight sites across Japan. | A posteriori: PCA with varimax rotation of 18 food groups from an FFQ; five dietary patterns, including “Japanese diet including protein and minerals”. | Prevalent all-cause dementia, Alzheimer disease, vascular dementia, and WML volume. | For the Japanese diet including protein and minerals pattern, adjusted Q4 versus Q1 ORs were 0.56 (95% CI 0.39–0.79) for all-cause dementia, 0.47 (0.32–0.69) for Alzheimer disease, and 1.02 (0.49–2.09) for vascular dementia. Higher pattern scores were also associated with lower WML volume. | Cross-sectional design; meta-analysis and visualization required longitudinal data. |

Note. The 25 reports in this table were retained in the review and synthesized qualitatively. Identifiers S1–S25 correspond to the supplementary references for the reports listed in Supplementary Tables 6 and 7. The final column records the report-level reasons for qualitative synthesis only. The requirements for inclusion in the meta-analyses and food-group loading visualizations are summarized in Supplementary Table 4. Reports evaluating both a priori and a posteriori dietary patterns are listed once in section A. “Both” indicates men and women. Models, comparison directions, and subgroups are retained as reported. Confidence intervals are 95% intervals. Risk-of-bias judgments for these 25 reports are presented in Supplementary Table 7. Applicable domains were assessed for cross-sectional reports, with no judgment assigned for Domain 4 or overall risk of bias.

Source notes. For Nakamura (2009), n = 9,086 follows the Abstract and Methods; some original table headings give 9,089. For Kumagai (1999), n = 630 follows the Methods and Table 1; inconsistent sex-specific counts elsewhere in the article were not reproduced. For Sugawara (2015), the outcome definition follows the Methods and Results, rather than the inconsistent headings/footnotes in Tables 2 and 4.

Abbreviations: AOR, adjusted odds ratio; AWGS, Asian Working Group for Sarcopenia; BDHQ, brief-type self-administered diet history questionnaire; BMI, body mass index; CI, confidence interval; CVD, cardiovascular disease; DBP, diastolic blood pressure; FA, factor analysis; FFQ, food frequency questionnaire; HDL, high-density lipoprotein; HR, hazard ratio; hs-CRP, high-sensitivity C-reactive protein; J-ECOH, Japan Epidemiology Collaboration on Occupational Health; JACC, Japan Collaborative Cohort Study; JDI, Japanese Diet Index; JPHC, Japan Public Health Center-based Prospective Study; JPSC-AD, Japan Prospective Studies Collaboration for Aging and Dementia; LDL, low-density lipoprotein; LTCI, long-term care insurance; MMSE, Mini-Mental State Examination; MoCA-J, Japanese version of the Montreal Cognitive Assessment; NILS-LSA, National Institute for Longevity Sciences–Longitudinal Study of Aging; OR, odds ratio; PCA, principal component analysis; PR, prevalence ratio; RR, relative risk (the original report’s label); RRR, reduced rank regression; SBP, systolic blood pressure; WML, white matter lesion.

**Supplementary Table 7. Risk-of-bias summary for studies included in the qualitative reviews.**

| **ID** | **Report** | **Design** | **D1 Confounding** | **D2 Exposure measurement** | **D3 Participant selection** | **D4 Post-exposure interventions** | **D5 Missing data** | **D6 Outcome measurement** | **D7 Selection of the reported result** | **Overall assessment** |
| --- | --- | --- | --- | --- | --- | --- | --- | --- | --- | --- |
| S1 | Nakamura 2009 | Longitudinal | High risk | High risk | Low risk | Low risk | Low risk | Low risk | Some concerns | High risk |
| S2 | Tomata 2014 | Longitudinal | High risk | Some concerns | Low risk | Low risk | High risk (PCA) Some concerns (JDI) | Low risk | Some concerns | High risk |
| S3 | Tomata 2016 | Longitudinal | High risk | Some concerns | Some concerns | Low risk | Some concerns | Low risk | Some concerns | High risk |
| S4 | Matsuyama 2020 | Longitudinal | High risk | Some concerns | Some concerns | Low risk | High risk | Low risk | Some concerns | High risk |
| S5 | Suthuvoravut 2020 | Cross-sectional | Some concerns | Some concerns | Some concerns | - | High risk | Low risk | Some concerns | - |
| S6 | Matsuyama 2021 | Longitudinal | Some concerns | Some concerns | Low risk | Low risk | Some concerns | Low risk | Some concerns | Some concerns |
| S7 | Lu 2021 | Longitudinal | High risk | Some concerns | Some concerns | Low risk | High risk | Low risk | Some concerns | High risk |
| S8 | Saji 2022 | Cross-sectional | High risk | High risk | High risk | - | Some concerns | Some concerns | Some concerns | - |
| S9 | Zhang 2023 | Longitudinal | Some concerns | Some concerns | Some concerns | Low risk | Low risk | Low risk | Some concerns | Some concerns |
| S10 | Miyake 2025 | Cross-sectional | Some concerns | Some concerns | Low risk | - | Low risk | Low risk | Some concerns | - |
| S11 | Kumagai 1999 | Longitudinal | Some concerns | High risk | Low risk | Low risk | Some concerns | Low risk | Some concerns | High risk |
| S12 | Sadakane 2008 | Cross-sectional | Some concerns | Some concerns | Some concerns | - | High risk | Low risk | Some concerns | - |
| S13 | Monma 2010 | Longitudinal | High risk | Some concerns | Low risk | Low risk | Low risk | Low risk | Some concerns | High risk |
| S14 | Ozawa 2013 | Longitudinal | Some concerns | Some concerns | Low risk | Low risk | Low risk | Low risk | High risk | High risk |
| S15 | Sugawara 2015 | Cross-sectional | Some concerns | Some concerns | Some concerns | - | No information | Low risk | Some concerns | - |
| S16 | Niu 2016 | Longitudinal | High risk | Some concerns | Some concerns | Low risk | Some concerns | Low risk | Some concerns | High risk |
| S17 | Shin 2016 | Longitudinal | Some concerns | Some concerns | Low risk | Low risk | Some concerns | Low risk | Some concerns | Some concerns |
| S18 | Okada 2016 | Longitudinal | Some concerns | Some concerns | Low risk | Low risk | Some concerns | Low risk | Some concerns | Some concerns |
| S19 | Okubo 2017 | Cross-sectional | Some concerns | Some concerns | Some concerns | - | Some concerns | Low risk | Some concerns | - |
| S20 | Kojima 2017 | Longitudinal | Some concerns | Some concerns | Some concerns | Low risk | Some concerns | Low risk | Some concerns | Some concerns |
| S21 | Htun 2017 | Cross-sectional | High risk | High risk | Some concerns | - | High risk | Low risk | Some concerns | - |
| S22 | Shin 2018 | Longitudinal | Some concerns | Some concerns | Low risk | Low risk | Some concerns | Some concerns | Some concerns | Some concerns |
| S23 | Zhao 2019 | Longitudinal | Some concerns | Some concerns | Low risk | Low risk | Some concerns | Low risk | Some concerns | Some concerns |
| S24 | Huang 2021 | Longitudinal | High risk | Some concerns | Low risk | Low risk | Some concerns | Some concerns | Some concerns | High risk |
| S25 | Chen 2026 | Cross-sectional | Some concerns | Some concerns | Some concerns | - | High risk | Low risk (dementia) Some concerns (WML) | Some concerns | - |

Note. Risk of bias was assessed using the review-specific operational criteria shown in Supplementary Table 1. For the 25 reports included in the qualitative synthesis, the model or result summarized in Supplementary Table 6 was fixed as the assessment target, and bias was judged for interpretation of the overall dietary-pattern effect. When review of the source article left two adjacent judgments plausible, the higher-risk judgment was assigned. 'No information' was retained when the information required to distinguish risk categories was not reported. For cross-sectional studies, Domain 4 (post-exposure interventions during follow-up) was not applicable and is shown as '-'; no Overall judgment was assigned, and Overall is also shown as '-'. For longitudinal studies, Overall corresponds to the highest risk-of-bias judgment across applicable domains. Reasons for non-inclusion in the analytic synthesis were not, by themselves, used as risk-of-bias criteria.

**Supplementary references for Supplementary Tables 6 and 7**

[S1] Nakamura Y, Ueshima H, Okamura T, Kadowaki T, Hayakawa T, Kita Y, Abbott RD, Okayama A. A Japanese diet and 19-year mortality: national integrated project for prospective observation of non-communicable diseases and its trends in the aged, 1980. British Journal of Nutrition. 2009;101(11):1696-705. doi: 10.1017/S0007114508111503.

[S2] Tomata Y, Watanabe T, Sugawara Y, Chou WT, Kakizaki M, Tsuji I. Dietary patterns and incident functional disability in elderly Japanese: the Ohsaki Cohort 2006 study. The Journals of Gerontology, Series A: Biological Sciences and Medical Sciences. 2014;69(7):843-51. doi: 10.1093/gerona/glt182.

[S3] Tomata Y, Sugiyama K, Kaiho Y, Honkura K, Watanabe T, Zhang S, Sugawara Y, Tsuji I. Dietary Patterns and Incident Dementia in Elderly Japanese: The Ohsaki Cohort 2006 Study. The Journals of Gerontology, Series A: Biological Sciences and Medical Sciences. 2016;71(10):1322-8. doi: 10.1093/gerona/glw117.

[S4] Matsuyama S, Zhang S, Tomata Y, Abe S, Tanji F, Sugawara Y, Tsuji I. Association between improved adherence to the Japanese diet and incident functional disability in older people: The Ohsaki Cohort 2006 Study. Clinical Nutrition. 2020;39(7):2238-2245. doi: 10.1016/j.clnu.2019.10.008.

[S5] Suthuvoravut U, Takahashi K, Murayama H, Tanaka T, Akishita M, Iijima K. Association between Traditional Japanese Diet Washoku and Sarcopenia in Community-Dwelling Older Adults: Findings from the Kashiwa Study. The Journal of Nutrition, Health & Aging. 2020;24(3):282-289. doi: 10.1007/s12603-020-1318-3.

[S6] Matsuyama S, Sawada N, Tomata Y, Zhang S, Goto A, Yamaji T, Iwasaki M, Inoue M, Tsuji I, Tsugane S, for the Japan Public Health Center-based Prospective Study Group. Association between adherence to the Japanese diet and all-cause and cause-specific mortality: the Japan Public Health Center-based Prospective Study. European Journal of Nutrition. 2021;60(3):1327-1336. doi: 10.1007/s00394-020-02330-0.

[S7] Lu Y, Matsuyama S, Sugawara Y, Sone T, Tsuji I. Changes in a specific dietary pattern and incident dementia: A prospective cohort study. Clinical Nutrition. 2021;40(5):3495-3502. doi: 10.1016/j.clnu.2020.11.036.

[S8] Saji N, Tsuduki T, Murotani K, Hisada T, Sugimoto T, Kimura A, Niida S, Toba K, Sakurai T. Relationship between the Japanese-style diet, gut microbiota, and dementia: A cross-sectional study. Nutrition. 2022;94:111524. doi: 10.1016/j.nut.2021.111524.

[S9] Zhang S, Otsuka R, Nishita Y, Tange C, Tomida M, Ando F, Shimokata H, Arai H. Twenty-year prospective cohort study of the association between a Japanese dietary pattern and incident dementia: the NILS-LSA project. European Journal of Nutrition. 2023;62(4):1719-1729. doi: 10.1007/s00394-023-03107-x.

[S10] Miyake H, Nanri A, Okazaki H, Miyamoto T, Kochi T, Kabe I, Tomizawa A, Yamamoto S, Konishi M, Inoue Y, Dohi S, Mizoue T. Close adherence to the Japanese diet is not associated with a high prevalence of hypertension: the Japan Epidemiology Collaboration on Occupational Health Study. British Journal of Nutrition. 2025;133(10):1342-1349. doi: 10.1017/S0007114525000844.

[S11] Kumagai S, Shibata H, Watanabe S, Suzuki T, Haga H. Effect of food intake pattern on all-cause mortality in the community elderly: a 7-year longitudinal study. The Journal of Nutrition, Health & Aging. 1999;3(1):29-33.

[S12] Sadakane A, Tsutsumi A, Gotoh T, Ishikawa S, Ojima T, Kario K, Nakamura Y, Kayaba K. Dietary patterns and levels of blood pressure and serum lipids in a Japanese population. Journal of Epidemiology. 2008;18(2):58-67. doi: 10.2188/jea.18.58.

[S13] Monma Y, Niu K, Iwasaki K, Tomita N, Nakaya N, Hozawa A, Kuriyama S, Takayama S, Seki T, Takeda T, Yaegashi N, Ebihara S, Arai H, Nagatomi R, Tsuji I. Dietary patterns associated with fall-related fracture in elderly Japanese: a population based prospective study. BMC Geriatrics. 2010;10:31. doi: 10.1186/1471-2318-10-31.

[S14] Ozawa M, Ninomiya T, Ohara T, Doi Y, Uchida K, Shirota T, Yonemoto K, Kitazono T, Kiyohara Y. Dietary patterns and risk of dementia in an elderly Japanese population: the Hisayama Study. The American Journal of Clinical Nutrition. 2013;97(5):1076-82. doi: 10.3945/ajcn.112.045575.

[S15] Sugawara N, Yasui-Furukori N, Umeda T, Tsuchimine S, Kaneda A, Tsuruga K, Iwane K, Okubo N, Takahashi I, Kaneko S. Relationship between dietary patterns and cognitive function in a community-dwelling population in Japan. Asia-Pacific Journal of Public Health. 2015;27(2):NP2651-60. doi: 10.1177/1010539513490194.

[S16] Niu K, Momma H, Kobayashi Y, Guan L, Chujo M, Otomo A, Ouchi E, Nagatomi R. The traditional Japanese dietary pattern and longitudinal changes in cardiovascular disease risk factors in apparently healthy Japanese adults. European Journal of Nutrition. 2016;55(1):267-79. doi: 10.1007/s00394-015-0844-y.

[S17] Shin S, Saito E, Inoue M, Sawada N, Ishihara J, Takachi R, Nanri A, Shimazu T, Yamaji T, Iwasaki M, Sasazuki S, Tsugane S, for the JPHC Study Group. Dietary pattern and breast cancer risk in Japanese women: the Japan Public Health Center-based Prospective Study (JPHC Study). British Journal of Nutrition. 2016;115(10):1769-1779. doi: 10.1017/S0007114516000684.

[S18] Okada E, Nakamura K, Ukawa S, Sakata K, Date C, Iso H, Tamakoshi A. Dietary Patterns and Risk of Esophageal Cancer Mortality: The Japan Collaborative Cohort Study. Nutrition and Cancer. 2016;68(6):1001-9. doi: 10.1080/01635581.2016.1192202.

[S19] Okubo H, Inagaki H, Gondo Y, Kamide K, Ikebe K, Masui Y, Arai Y, Ishizaki T, Sasaki S, Nakagawa T, Kabayama M, Sugimoto K, Rakugi H, Maeda Y, SONIC Study Group. Association between dietary patterns and cognitive function among 70-year-old Japanese elderly: a cross-sectional analysis of the SONIC study. Nutrition Journal. 2017;16(1):56. doi: 10.1186/s12937-017-0273-2.

[S20] Kojima R, Okada E, Ukawa S, Mori M, Wakai K, Date C, Iso H, Tamakoshi A. Dietary patterns and breast cancer risk in a prospective Japanese study. Breast Cancer. 2017;24(1):152-160. doi: 10.1007/s12282-016-0689-0.

[S21] Htun NC, Suga H, Imai S, Shimizu W, Takimoto H. Food intake patterns and cardiovascular risk factors in Japanese adults: analyses from the 2012 National Health and nutrition survey, Japan. Nutrition Journal. 2017;16(1):61. doi: 10.1186/s12937-017-0284-z.

[S22] Shin S, Saito E, Sawada N, Ishihara J, Takachi R, Nanri A, Shimazu T, Yamaji T, Iwasaki M, Sasazuki S, Inoue M, Tsugane S. Dietary patterns and prostate cancer risk in Japanese: the Japan Public Health Center-based Prospective Study (JPHC Study). Cancer Causes & Control. 2018;29(6):589-600. doi: 10.1007/s10552-018-1030-3.

[S23] Zhao W, Ukawa S, Okada E, Wakai K, Kawamura T, Ando M, Tamakoshi A. The associations of dietary patterns with all-cause mortality and other lifestyle factors in the elderly: An age-specific prospective cohort study. Clinical Nutrition. 2019;38(1):288-296. doi: 10.1016/j.clnu.2018.01.018.

[S24] Huang CH, Martins BA, Okada K, Matsushita E, Uno C, Satake S, Kuzuya M. A 3-year prospective cohort study of dietary patterns and frailty risk among community-dwelling older adults. Clinical Nutrition. 2021;40(1):229-236. doi: 10.1016/j.clnu.2020.05.013.

[S25] Chen L, Tatewaki Y, Thyreau B, Uchida K, Iki H, Nakaji S, Maeda T, Ono K, Noguchi-Shinohara M, Mimura M, Nakashima K, Iga JI, Takebayashi M, Ninomiya T, Taki Y. From the plate to the brain: associations between dietary patterns and reduced dementia prevalence and white matter lesions in older Japanese adults. GeroScience. 2026;48(2):2743–2756. doi: 10.1007/s11357-025-01791-7. Published online in 2025.
